# Unraveling the role of GPCR signaling in metabolic reprogramming and immune microenvironment of lung adenocarcinoma: a multi-omics study with experimental validation

**DOI:** 10.3389/fimmu.2025.1606125

**Published:** 2025-06-06

**Authors:** Zhaoxuan Wang, Cheng Wang, Shilei Zhao, Chundong Gu

**Affiliations:** ^1^ Department of Thoracic Surgery, The First Affiliated Hospital of Dalian Medical University, Dalian, China; ^2^ Department of Thoracic Surgery, Xishan People’s Hospital Of Wuxi City, Wuxi Branch of Zhongda Hospital Southeast University, Wuxi, China

**Keywords:** GPCR signaling, lung adenocarcinoma, metabolic reprogramming, immune microenvironment, ADM, prognostic model

## Abstract

**Background:**

Lung adenocarcinoma (LUAD) is characterized by metabolic and immune heterogeneity, driving tumor progression and therapy resistance. While G protein-coupled receptors (GPCR) signaling is known to regulate metabolism and immunity in cancers, its role in LUAD remains poorly defined. This study explores the influence of GPCR signaling on LUAD metabolism and immune landscape.

**Methods:**

We performed non-negative matrix factorization (NMF) clustering of GPCR signaling genes in TCGA-LUAD cohort to identify distinct molecular subgroups. A prognostic model was developed based on GPCR signaling genes using least absolute shrinkage and selection operator (LASSO) analysis and Cox regression. Differentially expressed genes were analyzed for metabolic pathway enrichment and immune infiltration. In addition, key genes within GPCR signaling were identified and validated through functional assays.

**Results:**

NMF clustering based on GPCR signaling identified three subgroups in LUAD, with cluster 3 exhibiting poorer overall survival and significant enrichment in multiple prognostic associated metabolism pathways including purine, pyrimidine, glyoxylate and dicarboxylate metabolism. Then, we developed a GPCRscore prognostic model and validated across multiple cohorts, which effectively stratified LUAD patients into distinct risk groups. High-risk LUAD patients had an immunosuppressive microenvironment and activated metabolic reprogramming. ADM was identified as a key gene in the high-risk group, correlating with tumor stage, immune suppression, and resistance to immunotherapy. Clinically, ADM was highly expressed in tumor tissues and shows elevated concentrations in the peripheral blood of patients with advanced-stage LUAD. Subsequently, we demonstrated that knock-down of ADM in LUAD cells impaired their proliferation, migration, and invasion, while also reducing the angiogenic potential of endothelial cells *in vitro*. Adrenomedullin promoted LUAD progression in a murine metastasis model. Further, adrenomedullin inhibited CD8^+^ T cells proliferation, induced exhaustion, and impaired cytotoxic function. Finally, drug sensitivity and cell viability analysis showed LUAD patients with high levels of ADM exhibited sensitivity to the treatment of Staurosporine and Dasatinib.

**Conclusions:**

In summary, this study reveals the pivotal role of GPCR signaling particularly mediated by ADM in orchestrating metabolic reprogramming and immune modulation in LUAD. ADM emerges as a potential predictive biomarker and therapeutic target, offering valuable implications for optimizing strategies.

## Introduction

Lung adenocarcinoma (LUAD), the most prevalent subtype of non-small cell lung cancer (NSCLC), poses a significant clinical challenge worldwide due to its high incidence and poor prognosis (1). While therapeutic paradigms have evolved from conventional surgery and chemoradiation to precision strategies incorporating targeted therapies (e.g., EGFR/ALK/ROS1 inhibitors) (2) and immune checkpoint inhibitors (anti-PD-1/PD-L1 agents) (3), significant challenges remain in addressing therapeutic resistance and unfavorable prognosis. These challenges underscore the urgent need for improved predictive biomarkers and more effective treatment strategies in LUAD. LUAD exhibits significant metabolic heterogeneity and immune microenvironment complexity, which contribute to tumor progression and therapeutic resistance. Metabolic reprogramming, a hallmark of cancer, enables tumor cells to adapt to fluctuating energy demands and microenvironmental constraints, facilitating immune evasion and disease progression (4). Emerging evidence suggests that the G protein-coupled receptor (GPCR) signaling pathway plays a pivotal role in modulating both metabolic processes and immune responses in various cancers (5–7), yet its precise role in LUAD remains insufficiently elucidated.

GPCR constitute the largest superfamily of membrane receptors, characterized by a structure comprising seven transmembrane domains, an extracellular N-terminal region, and an intracellular C-terminal tail (8). They function as critical mediators of signal transduction, translating extracellular stimuli into intracellular signaling networks and activating complex downstream effectors. In cancer, aberrant activation of GPCR modulates a wide range of oncogenic processes, including tumor cell proliferation, migration, and invasion, regulation of immune responses, promotion of angiogenesis, metastatic survival, reprogramming in glucose and lipid metabolism, adaptation to oxidative stress, and remodeling of the tumor microenvironment (TME) (9–12). Specially, lysophosphatidic acid (LPA) and its G-protein-coupled receptors (Lpar1) promoted tumor cell proliferation and motility through the PI3K/AKT signaling pathways (13). Besides, GPCR-mediated signaling could shape the TME by modulating cytokine secretion, immune cell recruitment, and stromal interactions (14–16). For instance, CXCR4 signaling facilitates the recruitment of immunosuppressive myeloid-derived suppressor cells (MDSCs) and regulatory T cells (Tregs) (17), contributing to immune evasion (18). Similarly, GPR132 promotes macrophage polarization toward a tumor-promoting M2 phenotype, thereby enhancing an immunosuppressive milieu (19). Notably, GPCR constitute the most extensive family of druggable membrane receptors, with over 30% of Food and Drug Administration (FDA)-approved drugs acting through them (20). For instance, CXCR4 antagonists such as plerixafor have been evaluated in hematologic malignancies for their ability to disrupt tumor-stroma interactions and sensitize cancer cells to chemotherapy (21). Similarly, agents targeting protease-activated receptors (PARs), chemokine receptors (e.g., CCR5 and CXCR2), and endothelin receptors have demonstrated anti-tumor effects in preclinical studies and early-phase clinical trials (22). These findings underscore the untapped potential of GPCR as therapeutic targets in cancer and highlight the need for further translational research to develop GPCR-directed anti-cancer therapies. However, the interplay between GPCR-driven metabolic reprogramming and TME remodeling in LUAD is not fully understood, highlighting the need for a comprehensive investigation. Multi-omics approaches, including transcriptomics, single-cell sequencing, and pathological features, provide powerful tools to dissect the molecular mechanisms underlying LUAD progression. By integrating these datasets, we systematically identified GPCR-associated metabolic reprogramming and their impact on immune cell infiltration and function. Furthermore, experimental validation using *in vitro* and *in vivo* models is essential to confirm the functional relevance of GPCR signaling in metabolic-immune crosstalk.

In this study, we used GPCR signaling genes to explore heterogeneity in LUAD patients and established the robustness prognostic model using LASSO and Cox regression based on integration of multiple bulk RNA-seq cohorts. The high-risk LUAD patients showed tumor progression, poor prognosis, low immune infiltration level, and metabolic reprogramming. Furthermore, we found that ADM in GPCR signaling significantly associated with metabolic reprogramming, immunosuppression, and immunotherapy resistance. Subsequent functional assays validated that knock-down of ADM in LUAD cells impaired their proliferation, migration, and invasion, while also reducing the angiogenic potential of endothelial cells *in vitro*. Besides, adrenomedullin (coding gene ADM) aggravated LUAD progression in the mouse metastasis model, suppressed *ex vivo* CD8^+^ T cells proliferation and cytotoxic function. Our study provides new insight into GPCR in shaping the TME, highlighting its potential as a therapeutic target for improving outcomes in LUAD patients with poor prognosis and resistance to conventional treatments.

## Methods

### Publicly available data collection and processing

We acquired survival data from bulk RNA-seq datasets available from The Cancer Genome Atlas (TCGA) and Gene Expression Omnibus (GEO) databases: TCGA-LUAD and GSE31210 (23). RNA-seq dataset of LUAD for the East Asian ancestry cohort (EAS) was obtained from Chen et al.’s study (24). Inclusion criteria for all cohorts included: a. histologically confirmed LUAD diagnosis; b. available gene expression data (microarray or RNA-seq); c. complete overall survival (OS). Exclusion criteria included: a. incomplete clinical or survival data; b. presence of other cancer types or mixed histology; and c. non-tumor samples. The level 4 RPPA dataset of LUAD patients including approximately 300 protein markers, covering all major cancer signaling pathways was retrieved from The Cancer Proteome Atlas (TCPA) (25). The spatial transcriptomics of one poorly differentiated LUAD sample was obtained from the study by Luo et al. (26). For microarray datasets, the normalization process was conducted using the R package “limma” (v3.54.1) (27). For bulk RNA-seq datasets, the public normalized gene expression data based on fragments per kilobase of exon model per million reads mapped (FPKM) was converted into Transcript Per Million (TPM), which were used as the gene expression matrix for downstream analysis. The gene-spot matrices generated after spatial transcriptomics data processing were analyzed with R package “Seurat” (v4.3.0) (28). Briefly, we analyzed spatial transcriptomics beginning with the Load10X_Spatial function. We applied the SCTransform function to normalize the spots. Spatial feature expression plots were created using the SpatialFeaturePlot function in Seurat. In addition, R package “SpaCET” (29) deconvolution algorithm was used to accessorily identify malignant cells.

### NMF clustering

The bulk RNA-seq expression profiles of GPCR signaling-related genes from the TCGA-LUAD dataset were analyzed using non-negative matrix factorization (NMF) clustering. NMF clustering based on GPCR signaling pathway (REACTOME_SIGNALING_BY_GPCR) were downloaded from the Molecular Signatures Database (MSigDB, https://www.gsea-msigdb.org/gsea/msigdb/). The R package “NMF” (30) was employed, with the number of clusters (rank) set between 2 and 6. Clustering was performed using the standard “lee” method with 100 iterations. The optimal number of clusters was determined based on the NMF rank survey metrics and the ability to effectively discriminate between distinct molecular subtypes.

### Construction of the GPCR risk score

The TCGA-LUAD cohort served as the primary training dataset, while GSE31210 and EAS cohorts were used as validation datasets. To identify prognostically relevant GPCR-related genes, we first performed univariate Cox proportional hazards regression analysis on the training cohort. Genes with a p-value < 0.05 were considered statistically significant and selected for further analysis. To reduce dimensionality and prevent model overfitting, we subsequently applied the least absolute shrinkage and selection operator (LASSO) regression using the “glmnet” R package. The optimal penalty parameter (λ) was determined via 10-fold cross-validation, and genes with non-zero coefficients at the optimal λ value were retained as candidate variables. Finally, the selected genes were subjected to multivariate Cox proportional hazards regression analysis to construct the prognostic model. The GPCR risk score was then calculated using the function “predict()” from the “glmnet” package. To assess the prognostic performance of the model, LUAD patients were stratified into high- and low-risk groups according to the median risk score. Overall survival (OS) differences between groups were analyzed using the log-rank test, while the predictive accuracy of model was evaluated through receiver operating characteristic (ROC) curve analysis.

### Immunological characteristics of the TME analysis

We employed a multi-faceted approach to evaluate the diversity in the TME within high GPCRscore and low GPCRscore of LUAD. Briefly, we utilized the ESTIMATE (31), EPIC (32), quantiseq (33), and TIMER (34) algorithms to elucidate the proportions of immune and stromal cells across histological subtypes. The above algorithms are included in the R package “IOBR” (v0.99.9) (35). Data on the use of deep learning to identify mappings of TILs from H&E pathological images of TCGA-LUAD were derived from the study by Saltz et al. (36).

### Identification of differentially expressed genes

The R package “DEseq2” (37) was used to identify differentially expressed genes (DEGs) for each modification pattern. An adjusted p-value < 0.05 and an absolute fold change > 2 were used as the criteria for the significance of DEGs.

### Functional gene set enrichment analysis

The over-representation analyses of Gene Ontology (GO) and Reactome pathways were performed using the R package “clusterProfiler” (v4.6.0) (38). Hallmark and Kyoto encyclopedia of genes and genomes (KEGG) gene sets were downloaded from MSigDB (https://www.gsea-msigdb.org/gsea/msigdb/). We performed Gene Set Enrichment Analysis (GSEA) (39) to examine the hallmark gene sets that are significantly enriched pathway.

### Drug sensitivity analysis

Drug sensitivity data were obtained from the Genomics of Drug Sensitivity in Cancer (GDSC2, https://www.cancerrxgene.org/), including GDSC2 gene expression profiles and corresponding drug response data. Ridge regression models were developed to assess the sensitivity of lung adenocarcinoma cells to 198 drugs. Lower half-maximal inhibitory concentration (IC50) values were indicative of heightened sensitivity to drug response. Using the R package “oncoPredict” (v1.2) (40), we calculated drug sensitivities within the TCGA-LUAD cohort.

### Cell lines and cell culture

Human lung adenocarcinoma cell lines (A549 and H1299) and primary human umbilical vein endothelial cells (HUVECs) were obtained from the American Type Culture Collection (ATCC). Cells were cultured in DMEM or RPMI-1640 medium supplemented with 10% fetal bovine serum (FBS), penicillin, and streptomycin. Small interfering RNAs (siRNAs) targeting ADM and corresponding negative controls were purchased from Sangon Biotech (Shanghai, China). A549 and H1299 cells were transiently transfected with siRNAs using Lipofectamine 2000 for 12 h, followed by functional and downstream assays. All cell lines were maintained at 37°C in a humidified incubator containing 5% CO_2_. The siRNA sequences are listed in **Supplementary Table S1**.

### RNA isolation, cDNA synthesis and qPCR

Total RNA was extracted using the UNIQ-10 Column RNA Isolation Kit, and reverse-transcribed with HiScript II Q RT SuperMix. Quantitative PCR was performed on an ABI ViiA7 system using ChamQ Universal SYBR qPCR Master Mix. GAPDH was used as the normalization control for the obtained results. Primer sequence were listed in **Supplementary Table S1**.

### Cell viability assays

To analyze the impact of ADM on LUAD cells proliferation, a total of 1×10^3^ A549 (si-NC and si-ADM) or H1299 cells (si-NC and si-ADM) were seeded in a 96-well plate and cultured in DMEM in humidified incubator for 24 h, 48 h, 72 h, and 96 h. The cells were incubated using Cell counting Kit-8 (CCK8) assay (HY-K0301, MCE) according to the manufacturer’s instructions. Besides, a total 3×10^3^ of A549 (si-NC and si-ADM) or H1299 (si-NC and si-ADM) cells were seeded in 6-well plate at 37°C, 5% CO_2_ in humidified incubator for 12 days. Colonies were fixed with 4% paraformaldehyde and stained with crystal violet solution. For drug sensitivity assays, the following compounds were used: Dasatinib (Selleck, S1021), Staurosporine (Selleck, S1421), and Zorifertinib (Selleck, S7971). Cells were seeded at a density of 3×10³ A549 cells per well in 96-well plates, and then treated with varying concentrations of each compound for 48 hours. Cell viability was then measured using the CCK8 assay.

### Migration and invasion assays

To study the cell migration of ADM on LUAD cells, a transwell migration assay was employed. Cells were subjected to an overnight fast in a medium containing only 1% FBS. Subsequently, 2×10^4^ A549 (si-NC and si-ADM) or H1299 cells (si-NC and si-ADM) (resuspended in 200 μl of FBS-free medium) were seeded into transwell chambers with an 8.0-μm pore-size membrane, placed in a 24-well plate filled with 30% FBS-containing media (600 μl). For invasion assays, the upper chamber was coated with 70 µl of matrix gel diluted at a ratio of 1:3 and incubated at 37°C for 30 minutes. After a 24-hour incubation for migration and invasion assays, cotton swabs were used to clean the interior of the insert, and the cells were fixed in 4% paraformaldehyde for 30 minutes, rinsed thrice with PBS, and stained with 0.5% crystal violet for 10 minutes. Images of cells were captured using a Zeiss microscope. Cellular migration was quantified by enumerating the nuclei of migrated cells.

### Conditioned medium collection and angiogenesis tube formation

A549 or H1299 (si-NC and si-ADM) cells were seeded in 60 mm dishes. When the cell confluence reached more than 90%, the medium was replaced with serum-free DMEM medium. The conditioned medium was collected after 48 h, then filtered through a 0.22 μm strainer, and diluted with DMEM with 20% FBS, at a ratio of 1:1. In a 96-well plate, 50 µl of Matrigel at a concentration of at least 10 mg/ml is added to each well and incubated at 37°C for 30 minutes to allow gelation. HUVEC cells pre-cultured with conditioned medium from A549 or H1299 (si-NC and si-ADM) for 48 h were resuspended in complete medium, and 100 µl of cell suspension containing 3 × 10^4^ cells was added to each well. The plate is then incubated for tube formation in the culture incubator, and images are captured at 2, 4, and 6-hour time points for observation. Quantitative analysis is performed using the Angiogenesis Analyzer plugin in Image J software.

### Murine CD8+ T cells isolation and T cells proliferation

Murine CD8^+^ T cells were isolated from the spleen of 6- to 8-week-old male C57BL/6 mice using the MojoSort Mouse CD8 T Cell Isolation Kit (480008, BioLegend) following the manufacturer’s instructions. Mouse T-Activator CD3/CD28 for T cell expansion and activation for 48 h (anti-mouse CD3, 5 μg/mL, 100238, BioLegend; anti-mouse CD28, 1 μg/mL, 102116, BioLegend). Murine CD8^+^ T cells cultured in RPMI-1640 medium supplemented with 10% FBS, 1% penicillin-streptomycin, and mouse IL-2 (200 ng/ml, PeproTech, 212-12-20UG). Then the same number of murine CD8^+^ T cells were cultured in the expansion media containing PBS or 200ng/ml adrenomedullin (LS-G11810, LSBio) for 24-72h, respectively.

### Flow cytometric analysis

Murine CD8^+^ T cells were cultured in the expansion media containing PBS or 200ng/ml adrenomedullin (LS-G11810, LSBio) and single-cell suspension were harvested. Cells were incubated with LIVE/DEAD FIX AQUA (L34966, Invitrogen, 1:200) or Fixable Viability Dye eFluor (65-0866-18, eBioscience, 1:200) and Fc receptor blocking reagent (553142, BD Pharmingen,1:400) on ice for 20 min and centrifuged at 400×g for 5 min. Then, cells were resuspended in the staining buffer and stained with antibodies on ice for 30 min in dark. Use the following antibodies: FITC anti-mouse CD8a (100705, BioLegend, 1:200); PE anti-human/mouse Granzyme B (372207, BioLegend, 1:200) Brilliant Violet 421™ anti-mouse CD366 (Tim-3) (119723, BioLegend, 1:200); APC anti-mouse CD279 (PD-1) (135209, BioLegend, 1:200). Flow cytometry was performed using an LSRFortessa instrument (BD Biosciences) and analyzed using FlowJo software (v10.8.1).

### Immunohistochemistry staining

Paraffin-embedded LUAD tissue samples were subjected to immunohistochemistry (IHC) to assess ADM protein expression. Briefly, tissues were first fixed in 10% formalin, dehydrated through graded ethanol, embedded in paraffin, and sectioned into 4μm-thick slices. Sections were deparaffinized in xylene and rehydrated through a descending ethanol series into distilled water. For antigen retrieval, the slides were heated in sodium citrate buffer (pH 6.0) using a microwave for 20 minutes. Endogenous peroxidase activity was quenched by incubating the slides with 3% hydrogen peroxide for 10 minutes at room temperature, followed by three washes with phosphate-buffered saline (PBS, pH 7.4). Non-specific binding was blocked with 3% bovine serum albumin (BSA) for 30 minutes. The slides were then incubated with the primary antibody against ADM (Proteintech, #10778-1-AP, 1:200) overnight at 4°C. After washing in PBS, sections were incubated with HRP-conjugated secondary antibodies (matched to the host species of the primary antibody) for 50 minutes at room temperature, followed by additional PBS washes. Color development was achieved using 3,3′-diaminobenzidine (DAB), which produced a brownish-yellow stain in positive areas. Hematoxylin was used as a counterstain to visualize nuclei. Slides were then dehydrated through a graded alcohol series (75%, 85%, two changes of 100%, and butanol, each for 5 minutes), cleared in xylene for 5 minutes, air-dried, and mounted with a resin-based medium. Positive cell detection was performed using QuPath software, with DAB as the chromogen and hematoxylin as the counterstain. The optical density (OD) thresholds were adjusted to classify cells into negative, weak (1+), moderate (2+), and strong (3+) positive staining intensities based on the DAB signal. The H-score was then automatically calculated within QuPath using the formula: H-score = (1 × %1+) + (2 × %2+) + (3 × %3+), yielding a final score ranging from 0 to 300 for each annotated tumor region.

### Adrenomedullin concentration detection

We prospectively collected peripheral blood samples from treatment-naïve patients with different clinical stages of LUAD from the First Affiliated Hospital of Dalian Medical University. The blood samples were collected using EDTA-treated tubes and centrifuged for 15 min at 1000g, and the plasma layer was transferred to separate tubes and stored at −80°C. Concentration of human adrenomedullin was detected by enzyme-linked immunosorbent assay (ELISA) kit (CUSABIO, CSB-E09146h) in accordance with the manufacturer’s instructions. Briefly, bring all reagents to room temperature before use for 30min and each well was then added with 100 μl of standard and test plasma, and incubated at 37°C for 2 h. Next, the liquid was removed, and 100 μl of biotin-labeled antibody (1x) was added to each well, followed by incubation at 37°C for 1 h. After washing the plate 3 times, 100 μl of horseradish peroxidase-labeled avidin working solution was added to each well, and incubated at 37°C for 1 h. After washing the plate 5 times, 90 μl of substrate was added to each well sequentially, and incubated at 37°C for 20 minutes for color development. The reaction was stopped by adding 50 μl of stop solution, and the optical density (OD value) of each well was measured at a wavelength of 450 nm.

### Animal experiments

The 6- to 8-week-old male C57BL/6 mice were purchased from Beijing Vital River Laboratory Animal Technology and housed in a specific pathogen-free environment with a 12/12h day/night cycle. Mice were used for construction of metastasis model with the 5.0 × 10^5^ LLC-luciferase cells through tail vein injection. Then, C57BL/6 mice were injected with PBS (n=6) or mouse recombinant adrenomedullin (n=5) (20 µg/kg) through tail vein every 3 days. *In vivo* imaging observation was performed 4 weeks after the implantation. IHC staining of anti-Ki-67 (#28074-1-AP, proteintech, 1:500) was used to detect metastatic LUAD cells. All animal maintenance and operational procedures were carried out in accordance with the animal ethical agreement (No.AEE24060) approved by the Animal Care and Ethics Committee of Dalian Medical University.

### Survival analysis

Survival analysis was performed using the R packages “survminer” (v3.1-8) and “survival” (v3.1-8). Patients within all datasets were divided into two groups based on the best-separation cut-off value determined by the ‘‘surv_cutpoint’’ function to plot the Kaplan–Meier survival curves, and P-value was calculated using a log-rank test. Univariate Cox proportional models were first used to analyze associations between the clinical parameters and OS, among which the parameters with statistical significance were further included in a multivariate Cox regression analysis. P-value < 0.05 was considered statistically significant.

### Statistical analyses

All statistical analyses and graphical presentations were performed using open-sourced R (v4.2.2) or GraphPad Prism software (v10.0). Quantification data are depicted as the mean ± standard deviation. As appropriate, statistical significance was determined using either Student’s t-test or Wilcoxon rank-sum test. Before the comparisons, the normality of the distributions was tested with the Shapiro-Wilk test. Correlation analysis was created with Pearson’s correlation. The statistical tests used in the figures are specified in the figure legends, and statistical significance was set at a P-value < 0.05. Significance levels are denoted as *P<0.05, **P<0.01, ***P<0.001, ****P<0.0001.

## Results

### GPCR-based NMF clustering reveals prognostic subtypes in TCGA-LUAD

**Figure 1:**
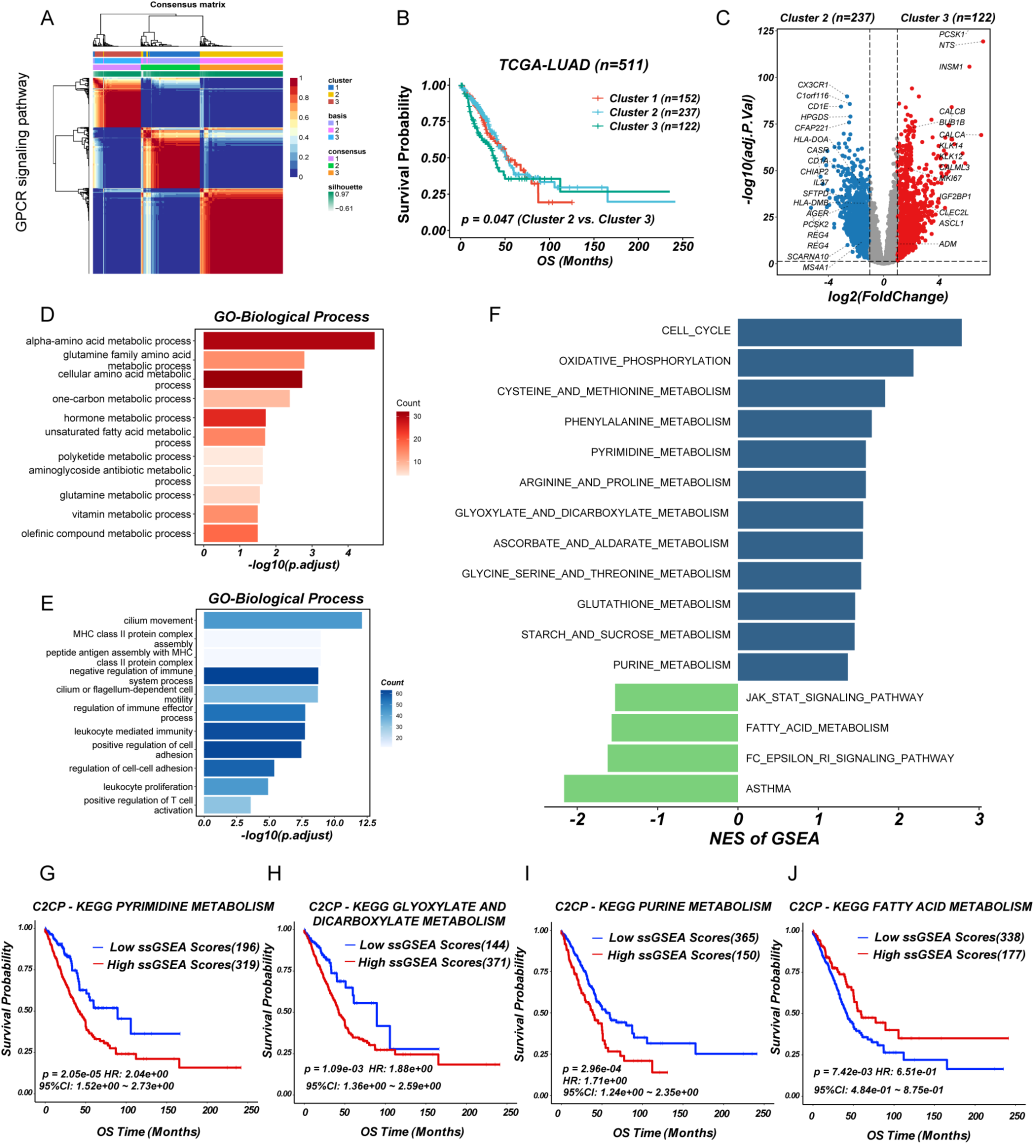
Identification of GPCR heterogeneity in LUAD patients. **(A)** Consensus matrix heatmap analysis. **(B)** Survival analysis of LUAD subgroups. **(C)** Volcano plot of differentially expressed genes between cluster 2 and 3. **(D, E)** Barplot showing the functional enrichment of using GO enrichment analysis. **(F)** Differential pathways were identified via GSEA analysis. **(G-J)** The enriched metabolism related pathway survival analysis based on ssGSEA score.

To investigate the role of GPCR signaling in LUAD, we performed non-negative matrix factorization (NMF) clustering based on GPCR signaling gene expression profiles (REACTOME_SIGNALING_BY_GPCR) from MSigDB using the TCGA-LUAD cohort. Based on the cophenetic correlation coefficient and dispersion, this analysis stratified LUAD patients into three distinct molecular subgroups, indicating heterogeneity in GPCR pathway activation (**Figure 1A**; **Supplementary Figure S1**). Notably, Kaplan-Meier survival analysis revealed that patients in cluster 3 exhibited significantly worse overall survival (OS) compared to cluster 2 (log-rank *P* < 0.05) (**Figure 1B**), suggesting that GPCR signaling may be associated with tumor aggressiveness and unfavorable clinical outcomes. To further explore the molecular differences among these subgroups, we conducted DEGs analysis, which identified a set of significantly upregulated (n=122) and downregulated (n=237) genes in cluster 3 compared to cluster 2 (**Figure 1C**). Gene ontology analysis showed that overexpressed genes in cluster 3 group involved in multiple metabolic pathways such as alpha-amino acid metabolic process, glutamine family amino acid metabolic process, and one-carbon metabolic process (**Figure 1D**), while cluster 2 group were related to immune-activating pathways including MHC class II protein complex assembly, leukocyte mediated immunity, and positive regulation of T cell activation (**Figure 1E**). In addition, GSEA based on KEGG pathways revealed distinct metabolic and signaling profiles across molecular subgroups. Specifically, cluster 3 was significantly enriched in pathways such as cell cycle, cysteine and methionine metabolism, purine metabolism, pyrimidine metabolism, as well as glyoxylate and dicarboxylate metabolism, whereas cluster 2 showed enrichment in the JAK-STAT signaling pathway and fatty acid metabolism (**Figure 1F**). Furthermore, prognostic analysis using the web-based tool PESSA (41) indicated that elevated activity of purine metabolism, pyrimidine metabolism, and glyoxylate and dicarboxylate metabolism had association with poor overall survival in LUAD patients, while enhanced fatty acid metabolism was correlated with favorable prognosis (**Figures 1G–J**). Given their enrichment in the high-risk subgroup, these metabolic alterations may contribute to the observed poor prognosis by supporting rapid tumor proliferation and influencing the TME. These findings suggested that GPCR-driven molecular subtypes in LUAD exhibit distinct metabolic and prognostic characteristics. Especially, the enrichment of nucleotide metabolism pathways in the high-risk group highlights a potential mechanistic link between GPCR signaling and metabolic reprogramming in LUAD, warranting further investigation into their functional relevance and therapeutic implications.

### Construction and validation of a prognostic model based on GPCR-related genes

**Figure 2:**
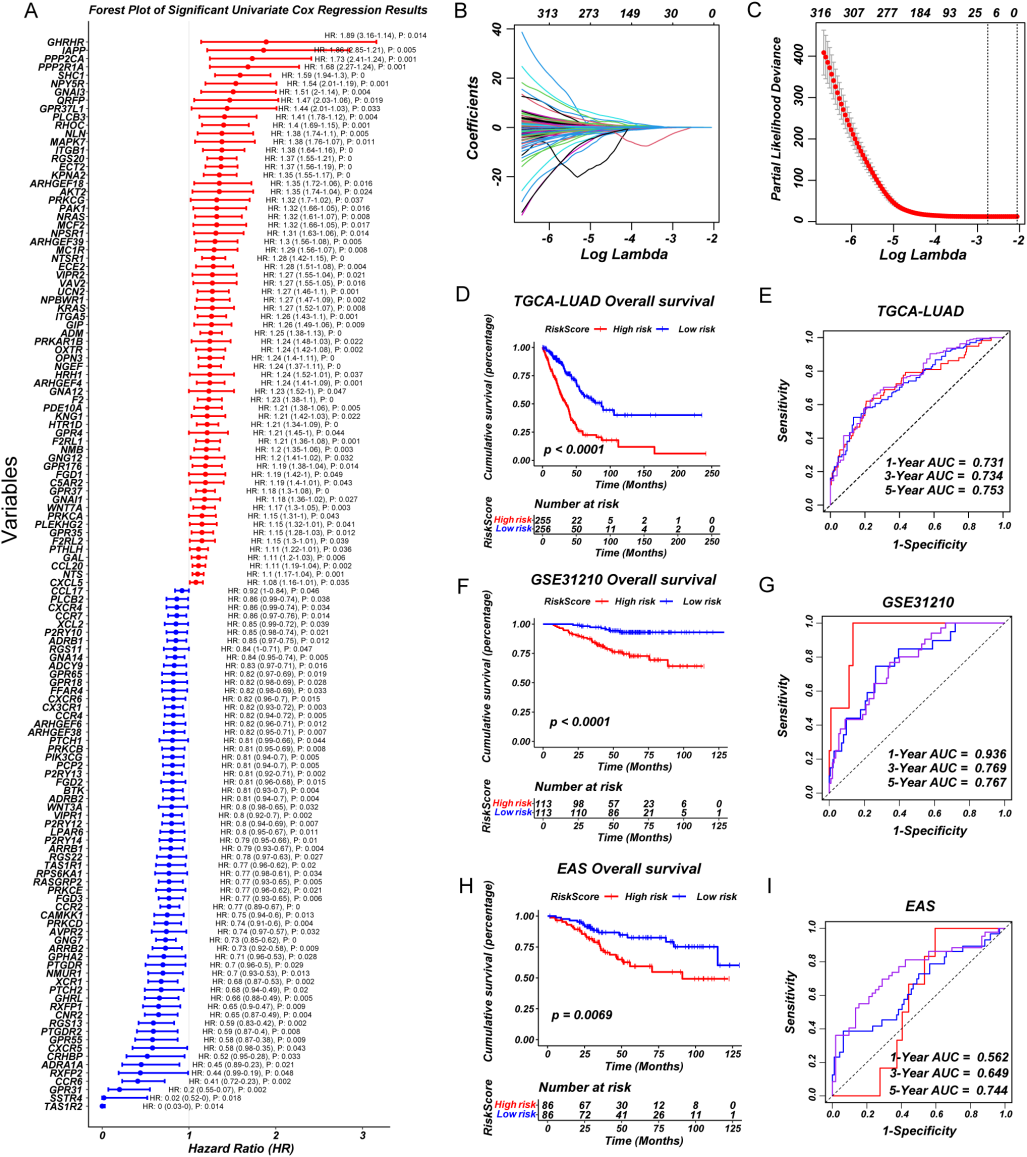
A consensus prognostic GPCRscore model was developed and validated. **(A)** Forest plot showing prognosis-associated GPCR signaling genes using univariate regression analysis. **(B, C)** Least absolute shrinkage and selection operator (LASSO) Cox regression were used to determine the optimal lambda and corresponding coefficients of the four indicators. **(D)** Kaplan–Meier curves of OS according to the GPCRscore in TCGA-LUAD (log-rank test: P <0.0001). **(E)** The diagnostic receiver operating characteristic (ROC) curve and time-related ROC curve confirmed the accuracy and stability of GPCRscore in predicting the prognosis of patients with TCGA-LUAD. **(F)** Kaplan–Meier curves of OS according to the GPCRscore in GSE31210 (log-rank test: P <0.0001). **(G)** The ROC curve and time-related ROC curve confirmed the accuracy and stability of GPCRscore in predicting the prognosis of patients with GSE31210. **(H)** Kaplan–Meier curves of OS according to the GPCRscore in EAS cohort (log-rank test: P =0.0069). **(I)** The ROC curve and time-related ROC curve confirmed the accuracy and stability of GPCRscore in predicting the prognosis of patients with EAS cohort.

Based on the significant heterogeneity in GPCR pathway activity observed across LUAD subgroups, we sought to develop a clinically relevant prognostic model. First, we performed univariate Cox regression analysis to identify GPCR-related genes significantly associated with OS (**Figure 2A**). To refine the selection and enhance model stability, we applied LASSO regression, reducing redundancy and preventing overfitting (**Figures 2B, C**). The final set of prognostic genes was further incorporated into a multivariate Cox regression model to construct a robust GPCR-based risk signature. To assess the predictive performance of our model, we validated it across multiple independent cohorts including GSE31210 and EAS cohort. The risk score effectively stratified patients into high- and low-risk groups based on median risk score, with the high-risk group consistently exhibiting significantly worse OS (*P* < 0.05) (**Figures 2D, F, H**). The distribution of GPCRscore and the survival status plot revealed that the high-risk group had higher GPCRscore and a greater proportion of deceased patients (**Supplementary Figures S2A-C**). Besides, time-dependent receiver operating characteristic (ROC) curve analysis measured the area under the curve (AUC) at 1-year, 3-year, and 5-year OS were 0.731, 0.734, and 0.753 in TCGA-LUAD, 1-year, 3-year, and 5-year OS were 0.936, 0.769, and 0.767 in GSE31210, 1-year, 3-year, and 5-year OS were 0.562, 0.649, and 0.744 in EAS cohort, respectively (**Figures 2E, G, I**). Multivariate Cox regression demonstrated that GPCRscore remained statistically significant (P < 0.05) after adjusting for available clinical traits, such as age, gender, and TNM stage, which suggested that GPCRscore was an independent risk factor for survival time `(**Supplementary Figures S2D-F**). Additionally, decision curve analysis (DCA) revealed that GPCRscore and TNM stage provided higher clinical benefit compared to other clinical features (**Supplementary Figures S2G-I**). These findings underscored the prognostic value of GPCR-related genes and highlight the clinical applicability of our risk model in LUAD prognosis.

### Transcriptomic, genomic profiling, and immune landscape differences between high- and low-risk groups

**Figure 3:**
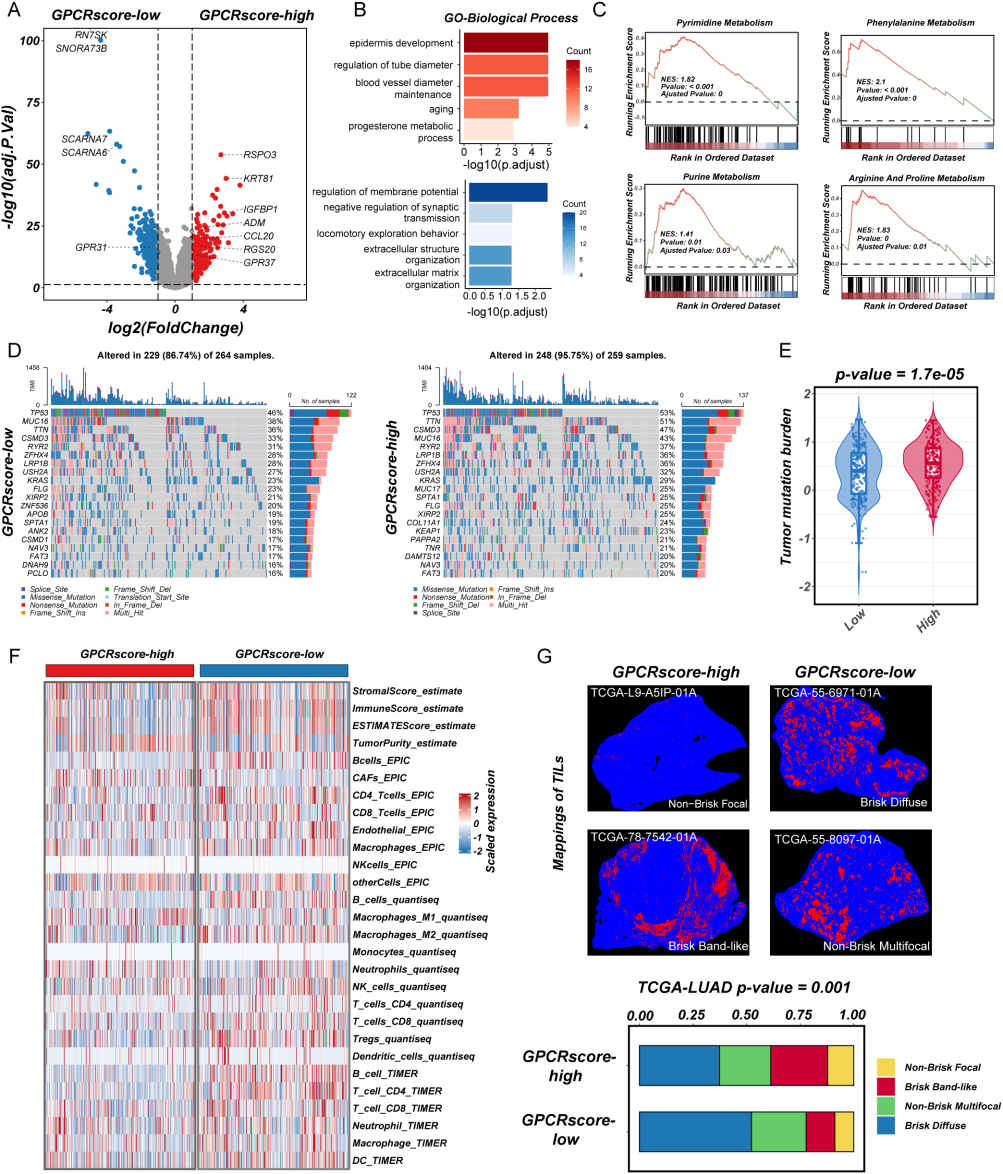
Transcriptional characteristics, genomic profiling, and immune infiltration in high-GPCRscore and low-GPCRscore LUAD patients. **(A)** The volcano plot of differential gene expressions in high-GPCRscore and low-GPCRscore group. The two vertical dashed lines represent absolute foldchange>2 in gene expression, and the horizontal dashed line denotes adjusted P-value cutoff 0.05. **(B)** Barplot showing the biological process of GO enrichment analyses in high- and low-GPCRscore group. **(C)** GSEA enrichment analyses of KEGG gene sets exhibited functional enrichment in high- and low-GPCRscore group. **(D)** Waterfall plot showing mutation profiles in the high- and low-GPCRscore group. **(E)** Violin plot illustrating the distribution of tumor mutational burden (TMB) across different risk groups. **(F)** Heatmap showing immune cell infiltration in the high- and low-GPCRscore group across four distinct computational methods including ESTIMATE, EPIC, quantiseq, and TIMER. **(G)** Representative staining of tumor-infiltrating lymphocytes from the H&E images in the high- and low-GPCRscore group. Barplot showing the GPCRscore across different mappings of TILs.

To investigate the molecular characteristics associated with GPCRscore, we first performed DEGs analysis between the GPCRscore-high and GPCRscore-low groups. As shown in the volcano plot (**Figure 3A**), multiple genes involved in immune regulation and metabolism, including *ADM*, *CCL20*, *IGFBP1*, *RSPO3*, and *RGS20*, were significantly upregulated in the GPCRscore-high group. In contrast, small nucleolar RNAs such as *SCARNA6* and *SNORA73B* were enriched in the GPCRscore-low group. GO enrichment analysis of the differentially expressed genes revealed significant enrichment in biological processes such as blood vessel diameter regulation, aging, regulation of membrane potential, and extracellular matrix organization (**Figure 3B**). To further explore functional pathway alterations, GSEA was performed and showed the GPCRscore-high group demonstrated significant enrichment in several metabolic pathways, including pyrimidine metabolism, purine metabolism, phenylalanine metabolism, and arginine and proline metabolism (**Figure 3C**). These results suggested that high GPCR activity is closely associated with reprogramming of nucleotide metabolism, potentially contributing to tumor progression and altered cellular functions.

Next, gene mutation analysis in the TCGA-LUAD cohort revealed a significantly higher frequencies of somatic mutations (95.75% vs. 86.74%) in GPCRscore-high group, including TP53 (53% vs. 46%), TTN (51% vs. 36%), CSMD3 (47% vs. 33%), MUC16 (43% vs. 38%), RYR2 (37% vs. 31%), and LRP1B (36% vs. 28%) (**Figure 3D**). In line with this observation, the GPCRscore-high tumors displayed significantly elevated levels of tumor mutational burden (TMB) (**Figure 3E**), suggesting a potential link between GPCR signaling activity and increased genomic instability.

To explore the immune-related characteristics of high- and low-risk groups, we conducted a comprehensive investigation into their underlying biological mechanisms. ESTIMATE analysis revealed that the high-risk group exhibited a higher tumor purity score but lower levels of stromal score, immune score, and overall ESTIMATE score. Subsequent analysis using multiple TME algorithms indicated that the GPCRscore-high group was characterized by an elevated cancer-associated fibroblasts (CAFs), while displaying lower levels of immune cell infiltration, particularly in B cells, T cells (CD8^+^ T cells and CD4^+^ T cells), and dendritic cells (**Figure 3F**). Moreover, using deep learning technology, as employed by Saltz et al. (37), we identified tumor-infiltrating lymphocyte (TIL) data in H&E-stained pathological images from the TCGA cohort. The subtype of TIL mapping and representative images confirmed that the “Brisk, band-like” and “Non-brisk, focal” infiltration patterns correlated with higher GPCRscore (**Figure 3G**), suggesting that the GPCRscore-high group is characterized by lower levels of lymphocyte infiltration. Even when immune cells do infiltrate, they form a band-like border around the tumor, which is insufficient to control tumor growth effectively within the tissue. Together, these findings suggested that the high-risk LUAD subgroup identified by our GPCR-based model is associated with nucleic acid metabolism activation and an immune-suppressive microenvironment, which may contribute to disease progression and poor clinical outcomes. These insights provided a potential rationale for targeting metabolic vulnerabilities and immune modulation in high-risk LUAD patients.

### ADM within GPCR-model linking metabolic reprogramming and immune suppression

**Figure 4:**
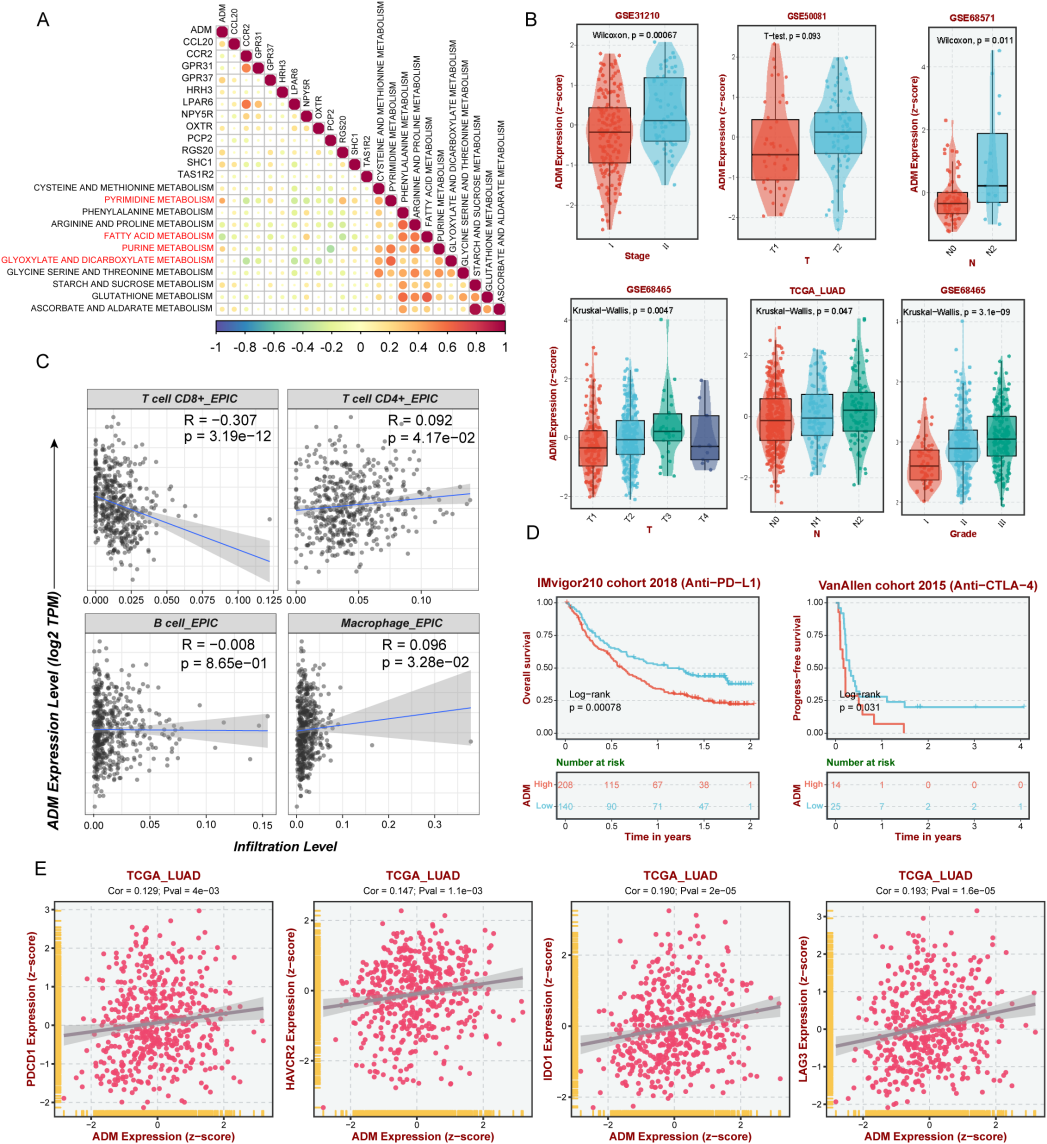
ADM as a key GPCR-associated gene linking metabolism and immune suppression. **(A)** Correlation heatmap showing the correlation between expression level of 13 genes in GPCRscore and metabolic enrichment score. **(B)** The expression of ADM in the different stage and pathological grade of LUAD patients. **(C)** Scatter plot showing the correlation between ADM and immune cells. **(D)** Survival analysis of ADM expression in immunotherapy cohorts. **(E)** Scatter plot showing the correlation between ADM and immunosuppressive checkpoint including *PDCD1*, *IDO1*, *HAVCR2*, and *LAG3*.

To identify potential mechanistic drivers within our prognostic model, we analyzed the correlation between the expression levels of the 13 model genes (*ADM*, *CCL20*, *CCR2*, *GPR31*, *GPR37*, *HRH3*, *LPAR6*, *NPY5R*, *OXTR*, *PCP2*, *RGS20*, *SHC1*, and *TAS1R2*) and key metabolic pathways. Among them, ADM exhibited positive association with metabolic reprogramming, particularly pyrimidine and purine metabolism and negative association with fatty acid metabolism, suggesting its potential role in tumor metabolic regulation (**Figure 4A**). Further clinical characteristics analysis revealed that ADM expression was significantly associated with tumor stage and pathological grade using BEST website (42) (**Figure 4B**), indicating its potential involvement in LUAD progression. Besides, estimation of the immune cell infiltrates showed a significantly negative correlation between CD8^+^ T cell infiltrates and ADM expression across TCGA-LUAD samples (**Figure 4C**). Additionally, a weaker correlation is observed between ADM expression and the infiltration of other immune cell types (**Figure 4C**). Further, ADM expression was found to be negatively correlated with immunotherapy benefit, with patients exhibiting high ADM expression showing worse prognosis with immune checkpoint blockade therapy including IMvigor210 (anti-PD-L1 therapy) and VanAllen cohort (anti-CTLA4 therapy) (**Figure 4D**). Consistently, we also found that ADM expression was positively correlated with multiple immune inhibitory checkpoints, including *PDCD1*, *HAVCR2*, *IDO1*, and *LAG3* further supporting its role in shaping an immunosuppressive TME and contributing to immune evasion in LUAD (**Figure 4E**). Together, these findings suggested that ADM may act as a key oncogenic driver that links metabolic reprogramming and immune suppression in LUAD, contributing to tumor progression and resistance to immunotherapy.

### ADM expression profiling in single-cell sequencing, clinical samples, and spatial transcriptomics

**Figure 5:**
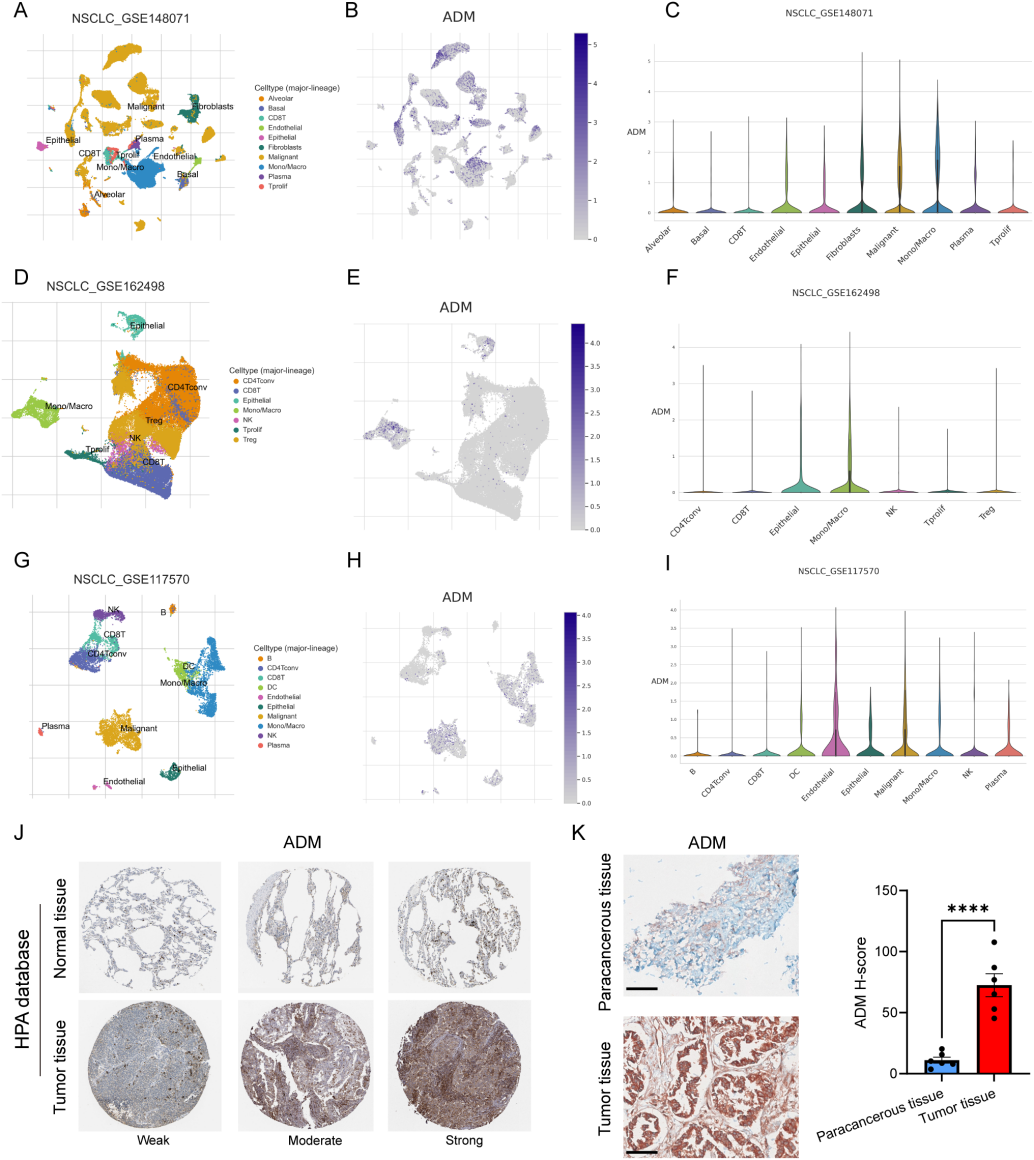
ADM expression in single-cell sequencing and pathological tissue. **(A)** UMAP plot showing cell types in GSE148071. **(B)** ADM expression in GSE148071. **(C)** Violin plot showing ADM expression in different cell types in GSE148071. **(D)** UMAP plot showing cell types in GSE162498. **(E)** ADM expression in GSE162498. **(F)** Violin plot showing ADM expression in different cell types in GSE162498. **(G)** UMAP plot showing cell types in GSE117570. **(H)** ADM expression in GSE117570. **(I)** Violin plot showing ADM expression in different cell types in GSE117570. **(J)** IHC staining of ADM in normal lung tissue and LUAD tissue from HPA database. **(K)** IHC staining of ADM in para-cancerous tissue (n=6) and LUAD tissue (n=6) from our center. The H-score showing the degree of positivity. Statistic tests: two-sided t test. Significance levels are denoted as *P<0.05, **P<0.01, ***P<0.001, ****P<0.0001. Scale bar: 100μm.

To further delineate the cellular sources and spatial distribution of ADM expression in LUAD, we performed integrative analysis using multiple single-cell RNA sequencing (scRNA-seq) datasets from the TISCH database (43), including GSE148071 (44), GSE162498 (45), and GSE117570 (46). Across these datasets, ADM was predominantly expressed in malignant epithelial tumor cells and tumor-associated macrophages (**Figures 5A–I**), rather than in stromal or lymphoid compartments. This dual expression pattern suggested that ADM may contribute to both tumor-intrinsic metabolic reprogramming and tumor-extrinsic immune modulation, aligning with its proposed role in shaping an immunosuppressive TME.

**Figure 6:**
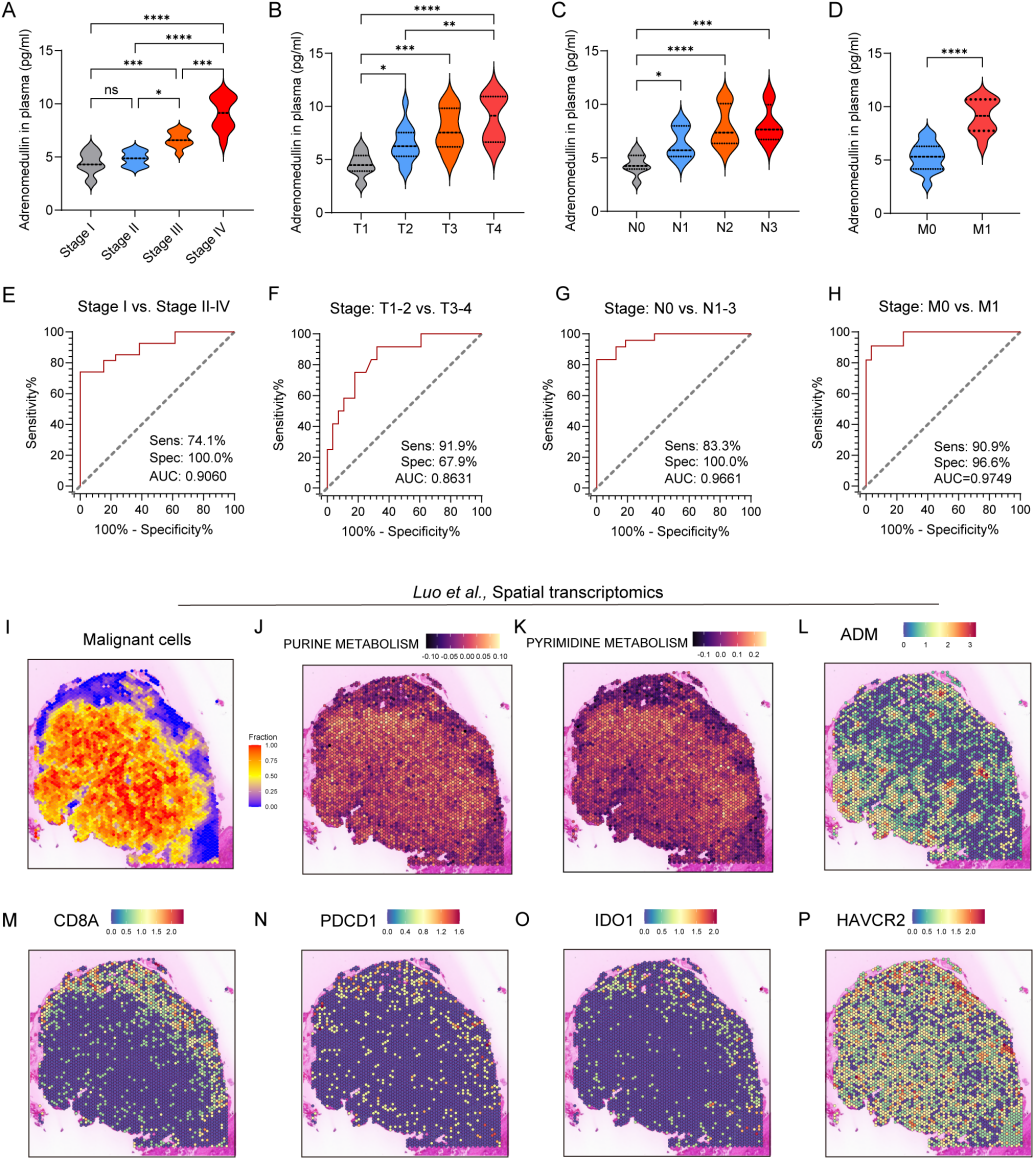
ADM expression in peripheral blood and spatial transcriptomics of LUAD patients. **(A–D)** Violin plot showing the level of adrenomedullin in different clinical stages **(A)**, stage T **(B)**, stage N **(C)**, and stage M **(D)** of LUAD patients. **(E–H)** ROC curves showing diagnostic efficiency to evaluate the sensitivity, specificity, and the area under the ROC curves (AUC) for differentiating different clinical stages **(E)**, stage T **(F)**, stage N **(G)**, and stage M **(H)** of LUAD patients. All ROC curve analyses were significant (p < 0.0001 from AUC of 0.5). **(I)** Spatial distribution of malignant cells inferred by SpaCET deconvolution. **(J, K)** Spatial enrichment of purine metabolism and pyrimidine metabolism pathway activity, demonstrating elevated metabolic activity in tumor-dense areas. **(L–P)** ADM, CD8A, PDCD1, IDO1, and HAVCR2 expression in spatial transcriptomics. Statistic tests: one-way ANOVA. Significance levels are denoted as *P<0.05, **P<0.01, ***P<0.001, ****P<0.0001.

To validate these findings at the protein level, we next examined ADM expression in immunohistochemical (IHC) staining images from the Human Protein Atlas (HPA) and in our own clinically collected LUAD specimens. Consistent with transcriptomic data, ADM protein expression was markedly elevated in LUAD tumor tissues compared to normal lung parenchyma and adjacent non-tumorous tissues (**Figures 5J, K**). In particular, ADM staining was localized to the cytoplasm of tumor cells and a subset of tumor-infiltrating immune cells, corroborating its cell-type specificity observed in scRNA-seq. Further, we prospectively collected a total of 40 plasma samples from LUAD patients. The level of plasma adrenomedullin in advanced-stage LUAD patients was significantly higher than in early-stage LUAD patients. Besides, we found that patients with T2-T4, N1-N3 and M1 exhibited significantly higher adrenomedullin levels compared with T1, N0 and M0, respectively (**Figures 6A–D**). In addition, ROC curve analyses to evaluate the diagnostic efficiency of adrenomedullin on clinical stage (Stage I vs. Stage II-IV), T stage (T1–2 vs. T3-4), N stage (N0 vs. N1-3), M stage (M0 vs. M1) with AUC of 0.9060 (Sensitivity=74.1%; Specificity=100.0%), 0.8631 (Sensitivity=91.9%; Specificity=67.9%), 0.9661 (Sensitivity=83.3%; Specificity=100.0%), and 0.9749 (Sensitivity=90.9%; Specificity=96.6%) (**Figures 6E–H**). These results emphasized the level of adrenomedullin was a potential peripheral blood marker to predict the occurrence of LUAD metastasis.

Next, spatial transcriptomics from the study by Luo et al. showed that pyrimidine and purine metabolism were upregulated in regions with dense malignant cell distribution (**Figures 6I-K**), suggesting active nucleotide biosynthesis in tumor cores. Notably, ADM expression showed strong spatial co-localization with metabolically active tumor regions, particularly those enriched in nucleotide metabolism (**Figure 6L**), implying a potential role for ADM in supporting tumor metabolic reprogramming. Furthermore, expression of immune markers associated with T cell cytotoxicity and exhaustion was evaluated. *CD8A* showed a scattered yet discernible presence in the peripheral tumor areas (**Figure 6M**). Exhaustion markers including *PDCD1*, *IDO1*, and *HAVCR2* were predominantly enriched in tumor-adjacent regions (**Figures 6N-P**), which exhibited partial spatial overlap with ADM expression. Collectively, these findings suggested that ADM was spatially associated with regions of heightened purine/pyrimidine metabolism and immune exhaustion, indicating its dual role in promoting tumor proliferation and immune evasion within TME of LUAD.

### ADM promotes tumor cells proliferation, migration, invasion, and pro-angiogenesis *in vitro* and *in vivo*

**Figure 7:**
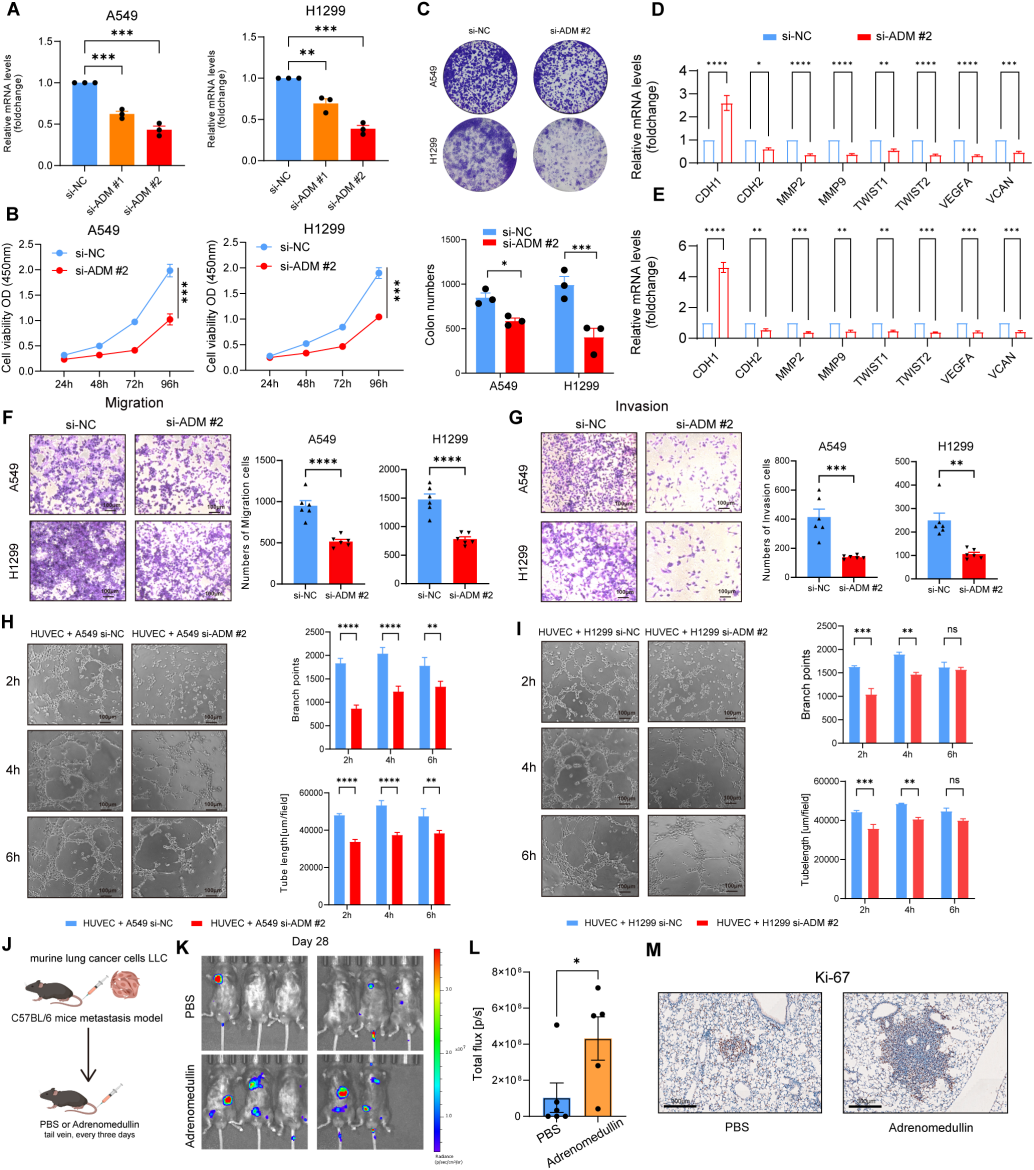
ADM promotes tumor cells proliferation, migration, invasion, and pro-angiogenesis *in vitro* and *in vivo.* **(A)** Barplot showing the mRNA level of ADM in LUAD cells (A549 and H1299) after si-ADM transfection. **(B)** Cell viability assay to evaluate the impact of si-ADM transfection on the proliferative ability of A549 and H1299 cells. **(C)** Colony formation assay to assess the impact of si-ADM transfection on the clonogenic capability of A549 and H1299 cells. **(D, E)** Barplot showing the mRNA level of CDH1, CDH2, MMP2, MMP9, TWIST1, TWIST2, VEGFA, VCAN in LUAD cells (A549 and H1299) after si-ADM transfection. **(F, G)** Transwell assay to evaluate the impact of si-ADM transfection on the migration and invasion ability of A549 and H1299 cells. **(H, I)** Endothelial tube-formation assay to evaluate the impact of si-ADM transfection on the promoting angiogenesis ability of A549 and H1299 cells. **(J)** *In vivo* LUAD tail vein injection model showing the effect of adrenomedullin on LUAD metastasis. **(K, L)** Fluorescence images and quantifications of metastatic lesions. **(M)** Ki-67 antibody was used to detect murine tumor cells. Statistic tests: two-sided t test. Significance levels are denoted as *P<0.05, **P<0.01, ***P<0.001, ****P<0.0001.

To validate the above findings, we knocked down ADM expression in A549 and H1299 cells using siRNA (**Figure 7A**). Cell viability was assessed using the CCK8 assay, which revealed that ADM inhibition significantly impaired the proliferative capacity of LUAD cells (**Figure 7B**). This was further corroborated by colony formation assays, which showed a similar trend (**Figure 7C**). In addition, significant decreased enrichment of EMT pathway (*CDH1, CDH2, MMP2, MMP9, TWIST1*, and *TWIST2*) and angiogenesis pathway (*VEGFA* and *VCAN*) were observed in knocked down ADM expression in A549 and H1299 cells (**Figures 7D, E**). Transwell migration and invasion assays demonstrated that knockdown of ADM notably reduced the migratory and invasive capabilities of LUAD cells (**Figures 7F-G**). Additionally, when HUVEC cells were co-cultured with A549 cells and H1299 cells transfected with si-ADM, a significant decrease in both branch points and tube length was observed (**Figures 7H-I**), suggesting that ADM plays a key role in promoting angiogenesis in the TME.

Next, we injected murine LLC-luciferase cells through the tail vein into immune-competent C57BL/6 mice to construct a metastasis model. Adrenomedullin recombinant protein was intravenously injected into immune-competent C57BL/6 mice every 3 days (**Figure 7J**). Bioluminescence imaging revealed a significant increase of fluorescence signal in the distant metastasis C57BL/6 mice injected with adrenomedullin (**Figures 7K-L**). Tumor cells (Ki-67^+^ cells) were detected in the lesion of intrapulmonary metastasis (**Figure 7M**), which exhibited a significant increase in lung metastasis compared to the control group. Collectively, these findings provided preclinical evidence supporting ADM as a pro-metastasis factor contributed to poor clinical outcomes for LUAD patients through promoting tumor neovascularization.

### ADM suppresses CD8+ T cell proliferation and induces exhaustion

**Figure 8:**
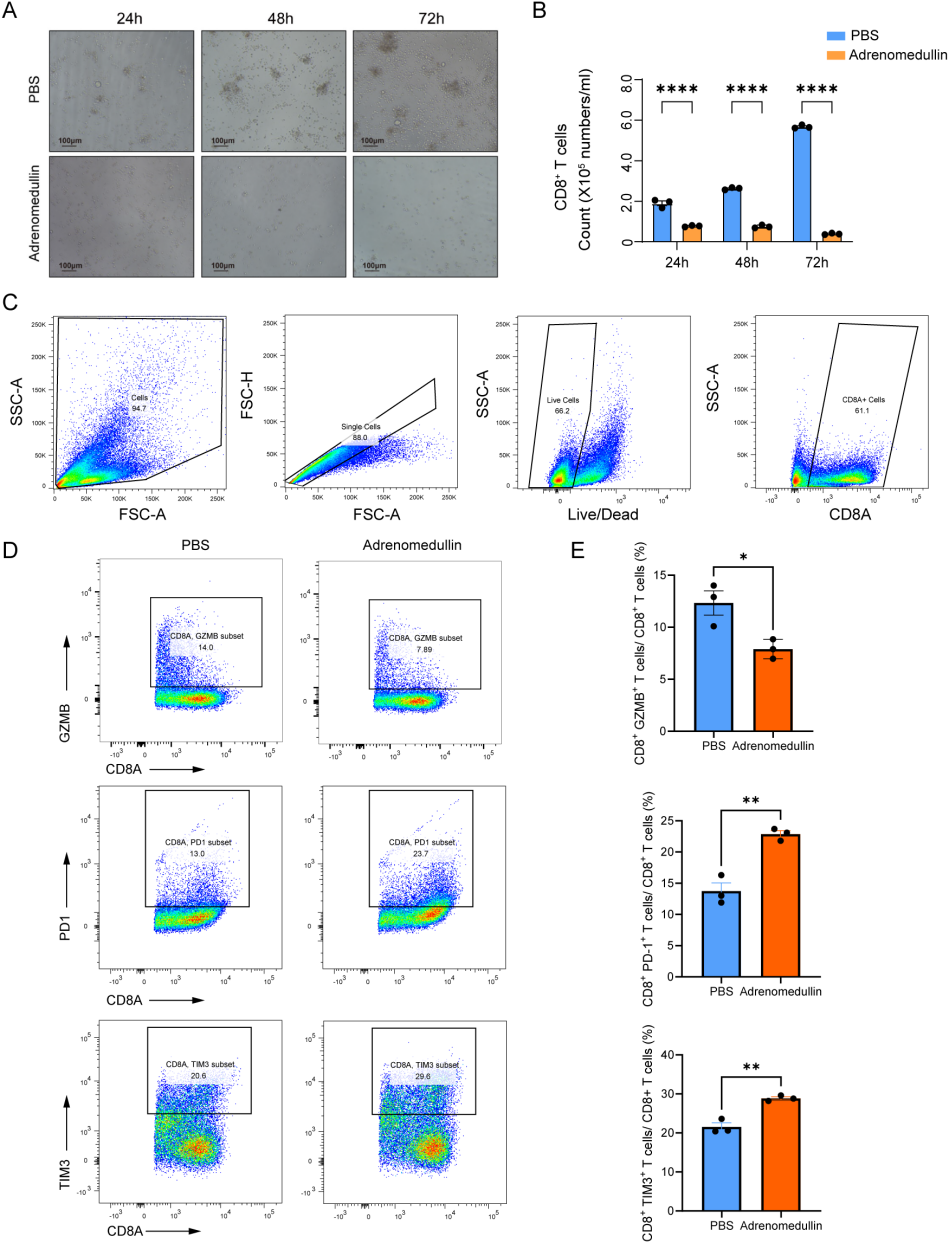
ADM suppresses CD8^+^ T cell proliferation and induces exhaustion. **(A)** Representative bright-field images of CD8^+^ T cells cultured with PBS or adrenomedullin (200ng/ml) at 24, 48, and 72 hours. **(B)** Quantification of CD8^+^ T cell numbers at 24, 48, and 72 hours. ADM significantly reduced proliferation compared to PBS control. **(C)** Gating strategy for flow cytometric identification of live CD8^+^ T cells from culture. **(D)** Representative flow cytometry showing expression of cytotoxicity marker GZMB and exhaustion markers PD-1 and TIM3 in CD8^+^ T cells following adrenomedullin (200ng/ml) or PBS treatment at 72 hours. **(E)** Quantification of GZMB^+^, PD-1^+^, and TIM3^+^ CD8^+^ T cells. Statistic tests: two-sided t test. Significance levels are denoted as *P<0.05, **P<0.01, ***P<0.001, ****P<0.0001.

To elucidate its effects, we isolated primary CD8**^+^** T cells from the spleens of C57BL/6 mice and cultured them in the presence of recombinant murine adrenomedullin protein (200 ng/mL). After 24 to 72 hours of incubation, *ex vivo* proliferation assays revealed a marked suppression of CD8^+^ T cell proliferation in adrenomedullin-treated groups compared to controls (**Figures 8A-B**), indicating that ADM directly impaired CD8**^+^** T cells expansion. Flow cytometry analysis further demonstrated that adrenomedullin induced an exhaustion phenotype in CD8**^+^** T cells (**Figure 8C**). Specifically, there was a significant upregulation of exhaustion-associated markers, including PD-1 and TIM3, alongside a concomitant downregulation of key cytotoxic effector molecules Granzyme B (GZMB) (**Figures 8D-E**). These findings suggested that ADM not only inhibits T cell proliferation but also actively drives functional exhaustion, thereby impairing their anti-tumor capacity. Taken together, these results highlighted ADM as a dual-function modulator that promoted tumor progression by simultaneously inducing immune suppression and enabling metastatic dissemination. Targeting ADM or its downstream signaling pathways may therefore offer a promising therapeutic strategy to restore CD8**^+^** T cells function and enhance anti-tumor immunity, particularly in metastatic or immunotherapy-resistant settings.

### ADM-associated metabolic reprogramming and drug sensitivity analysis

**Figure 9:**
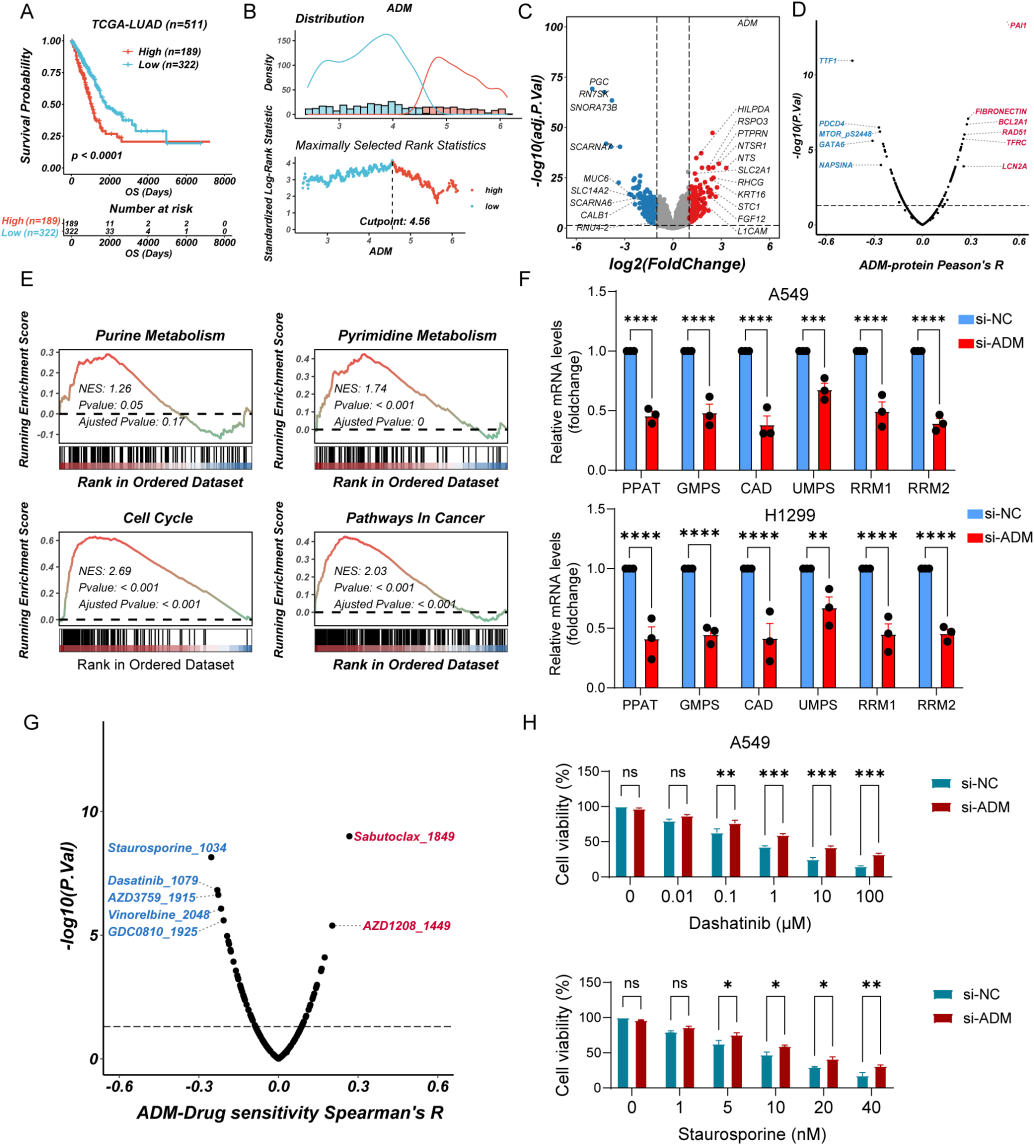
ADM-associated metabolic reprogramming and drug sensitivity analysis **(A)** Kaplan–Meier curves of OS according to ADM in TCGA-LUAD cohort (log-rank test: P <0.0001). **(B)** Threshold Justification of ADM in TCGA-LUAD. **(C)** Volcano plot of differentially expressed genes between high expression of ADM and Low expression of ADM. **(D)** The scatterplot showing the correlation between ADM and the RPPA protein level in TCGA-LUAD. The blue color indicates a significantly negative correlation (P-value < 0.05, Peason's R<-0.3), while the red color represents a significantly positive correlation (P-value < 0.05, Peason's R>0.3). **(E)** GSEA of DEGs showing the enrichment of tumor metabolic pathways. **(F)** Barplot showing the mRNA level of PPAT, GMPS, CAD, UMPS, RRM1, and RRM2 in LUAD cells (A549 and H1299) after si-ADM transfection. **(G)** The scatterplot showing the correlation between the GPCRscore and IC50 values for 198 compounds in TCGA-LUAD. The blue color indicates a significantly negative correlation (P-value < 0.05, Peason's R<-0.2), while the red color represents a significantly positive correlation (P-value < 0.05, Peason's R>0.2). **(H)** Barplot showing the relative viability of A549 cells treated with Staurosporine or Dasatinib after si-ADM transfection, in comparison with the control group at 48 hours. Statistic tests: two-way ANOVA. Significance levels are denoted as ns, P>0.05, *P<0.05, **P<0.01, ***P<0.001, ****P<0.0001.

To further explore the impact of ADM expression on tumor metabolism, we grouped LUAD patients using the best cutoff calculated by maximally selected rank statistics (**Figures 9A, B**). We analyzed DEGs between ADM-high and ADM-low expression groups (**Figure 9C**). Additionally, we conducted correlation analysis between the expression level of ADM and RPPA data. This analysis revealed a significant positive correlation between ADM and pro-metastatic proteins such as PAI-1. In contrast, the well-differentiated LUAD markers, TTF1 and Napsin A, showed a significant negative correlation with ADM (**Figure 9D**). GSEA revealed that the ADM-high group exhibited significant enrichment in pyrimidine metabolism, glycolysis, and lipid metabolism pathways, consistent with its role in promoting metabolic adaptation and tumor progression. In contrast, the ADM-low group showed enrichment in oxidative phosphorylation and immune activation pathways, suggesting a less aggressive metabolic phenotype (**Figure 9E**; **Supplementary Figure S3A**). Notably, correlation analysis found that ADM expression was positively correlated with multiple rate-limiting enzymes in both purine (*PPAT*, *GMPS*, *RRM1*, and *RRM2*) and pyrimidine (*CAD* and *UMPS*) metabolic pathways (**Supplementary Figure S3B**). To experimentally validate these findings, we performed RT-qPCR analysis following ADM knockdown in lung cancer cells. Conformably, we observed that the mRNA expression levels of above genes were significantly reduced upon ADM knockdown (**Figure 9F**), supporting a regulatory role of ADM in maintaining nucleotide biosynthesis gene expression.

To further explore the therapeutic relevance of ADM expression, we performed drug sensitivity analysis using pharmacogenomic databases. Specifically, oncoPredict was used to assess the sensitivity of 198 compounds from the GDSC2 database in TCGA-LUAD samples stratified by ADM expression levels. Spearman correlation analysis revealed that ADM expression was significantly negatively correlated with the predicted IC50 values of five compounds including Staurosporine, Dasatinib, AZD3759 (Zorifertinib), Vinorelbine, and GDC0810 (Spearman’s R < −0.2, *P* < 0.05) suggesting enhanced sensitivity in ADM-high tumors. Conversely, positive correlations were observed with Sabutoclax and AZD1208 (Spearman’s R > 0.2, *P* < 0.05), indicating potential resistance (**Figure 9G**). To validate these findings, we selected three candidate drugs (Staurosporine, Dasatinib, and Zorifertinib) and conducted cell viability assays in ADM-knockdown A549 cells versus control cells. Consistent with in silico predictions, low expression of ADM cells exhibited reduced sensitivity to all three compounds, with remarkable differences observed for Staurosporine and Dasatinib (**Figure 9H**; **Supplementary Figure S4**). Collectively, these results suggested that high ADM expression may serve as a potential biomarker for increased responsiveness to Staurosporine and Dasatinib in LUAD.

## Discussion

Given the distinct heterogeneity of LUAD, the prognosis of LUAD patients exhibits substantial variability. Aberrant GPCR activation influences key oncogenic pathways, including glucose and lipid metabolism, oxidative stress responses, and immune landscape. In this study, we identified three distinct molecular subgroups in LUAD, which revealed significant heterogeneity in GPCR pathway activation through NMF clustering. Then, we explored the role of GPCR signaling in LUAD by developing a prognostic model and examining its association with tumor metabolism and immune evasion. Our findings suggested that GPCR signaling played a pivotal role in LUAD progression, with certain subgroups exhibiting distinct metabolic and immune characteristics. Notably, high-risk patients, characterized by increased activation of purine and pyrimidine metabolism, was associated with poor prognosis and an immunosuppressive TME. The prognostic value of our GPCR-based model was further validated across multiple independent cohorts, with high-risk patients consistently exhibiting worse OS. This model, developed through a multivariate Cox regression approach and validated by time-dependent receiver operating characteristic (ROC) curves, highlights the clinical utility of GPCR-related genes in stratifying LUAD patients based on their risk of poor clinical outcomes.

A critical observation in this study was the identification of ADM as a key gene in our GPCR-related risk model. The calcitonin-like receptor (CLR) is a typical GPCR, which plays a role in regulating various physiological processes, including blood pressure, metabolism, and nervous system function. The signaling of CLR can be regulated through co-expression with one of three receptor activity-modifying proteins (RAMPs). RAMPs influence the binding of CLR with its endogenous ligands and determine the specific signaling pathway it activates (47). Specifically, when CLR is co-expressed with RAMPs, it can be activated by different endogenous ligands, including: calcitonin gene-related peptide (CGRP), ADM, and ADM2 (47). According to our analyses, ADM exhibited positive correlation with metabolic reprogramming, particularly in purine (*PPAT*, *GMPS*, *RRM1*, and *RRM2*) and pyrimidine (*CAD* and *UMPS*) metabolic pathways, suggested that it played an important role in tumor cell metabolic adaptation. As support, ADM could enhance PI3K (48) and MAPK signaling (49), which may indirectly upregulate rate-limiting enzymes such as CAD (50). Future studies are needed to further explore the mechanisms underlying ADM involvement in nucleotide metabolism. Moreover, ADM expression was linked to immune evasion, with higher expression levels correlating with reduced CD8^+^ T cell infiltration and resistance to immunotherapy. These findings underscored dual role of ADM in promoting both metabolic changes and immune suppression, positioning it as a potential therapeutic target in LUAD. Consistent with the known roles of adrenomedullin in tumor progression, our findings reinforced the notion that ADM functions not only as a pro-angiogenic and pro-survival factor but also as a key modulator of the TME. The significant upregulation of ADM in tumor tissues strengthened its potential as a biomarker for LUAD progression and its promise as a therapeutic target. Further, our *in vitro* and *in vivo* experiments provided compelling evidence that ADM contributed to tumor progression and metastasis. ADM inhibition suppressed LUAD cell proliferation, migration, invasion, and angiogenesis. Recombinant adrenomedullin (coding gene ADM) limited CD8^+^ T cell proliferation and cytotoxicity and promoted the exhaustion. These results aligned with the clinical findings, supporting ADM as a pro-metastatic factor and its involvement in immune escape mechanisms.

Previous studies have highlighted dysregulation of adrenomedullin in various types of tumors such as osteosarcomas, pancreatic cancer, prostate cancer, and gastric cancer (51–54). Mechanistically, ADM overexpression in cancer cells enhances neovascularization, thereby facilitating macroscopic metastatic outgrowth, particularly after initial entrapment in lymphatic vessels. In breast cancer, clinical evidence supports this notion, as higher ADM protein levels are significantly associated with axillary lymph node metastasis (55). Similarly, in colorectal cancer, ADM is among the most selectively upregulated genes in KRAS-mutant cells under hypoxia, and its expression is markedly reduced upon KRAS silencing, highlighting ADM as a key downstream effector of KRAS-driven tumorigenesis in hypoxic niches (56). Beyond its vascular functions, ADM also exerts potent immunomodulatory effects, notably by reprogramming TAMs toward an M2-like, immunosuppressive phenotype. This dual capacity to simultaneously drive angiogenesis and immune evasion may underlie the association of ADM with aggressive disease phenotypes across a spectrum of cancers, including glioma (57), ovarian (58), and prostate cancers (59). Furthermore, a study reported adrenomedullin promotes resistance to sunitinib, through the reduction of FDX1 expression levels, thus inhibiting cuproptosis in renal cell carcinoma (60). In addition, adrenomedullin also could induce cisplatin resistance in ovarian cancer through reprogramming of glucose metabolism (61). Taken together, these observations indicate ADM as a multifunctional player in the TME, coordinating vascular remodeling, immune suppression, and lineage plasticity in response to environmental pressures such as hypoxia and therapeutic stress. These findings not only highlight the clinical significance of ADM expression in predicting metastatic potential and treatment resistance but also support the rationale for therapeutic strategies targeting ADM signaling to disrupt tumor-supportive stromal remodeling and restore antitumor immunity.

Next, we aimed to determine whether certain therapeutic agents might be particularly effective in tumors with high ADM expression. Through bioinformatics-driven drug sensitivity analysis and experimental validation, we identified a strong association between elevated ADM levels and increased responsiveness to Staurosporine and Dasatinib. Staurosporine, a naturally derived compound from soil microorganisms, is a potent inhibitor of protein kinase C (PKC) and has long been recognized for its potential as an anti-cancer agent (62). In contrast, Dasatinib exhibits broader kinase inhibition, targeting not only Src family kinases but also several other tyrosine kinases (63). Despite their promising profiles, the clinical application of these tyrosine kinase inhibitors (TKIs) in solid tumors such as LUAD has been limited. This is likely due to the distinct molecular landscape of solid tumors compared to hematological cancers, as well as concerns over off-target effects and associated toxicity. The Src family kinases are key regulators of multiple signaling cascades, including the PI3K/AKT axis (64). Since ADM is known to activate the PI3K/AKT pathway (48), inhibition of Src kinases by agents like Dasatinib may disrupt ADM-mediated signaling. This mechanistic overlap may underlie the heightened sensitivity of ADM-high tumors to these inhibitors.

However, our study also highlights some important limitations and areas for future research. While we have established ADM as a key player in LUAD progression, its precise molecular mechanisms and interaction with other signaling pathways remain to be elucidated. Additionally, while our model shows promise in predicting patient prognosis, further validation in larger, diverse cohorts is required to confirm its clinical applicability. The potential for combining GPCR-related biomarkers, such as ADM, with immunotherapy or targeted therapies offers an exciting avenue for future studies. Moreover, further exploration of the relationship between metabolic reprogramming and immune modulation in LUAD could provide deeper insights into TME dynamics and therapeutic opportunities.

## Conclusion

In summary, our study identified a GPCR-driven molecular subtyping system for LUAD that not only stratifies patients based on prognosis but also reveals critical insights into tumor metabolism and immune suppression. The identification of ADM as a key mediator of metabolic reprogramming and immune evasion provided a valuable target for future therapeutic strategies. Moving forward, the integration of GPCR signaling modulation with metabolic and immune therapies could offer new approaches to improving patient outcomes in LUAD.

## Data Availability

The datasets presented in this study can be found in online repositories. The names of the repository/repositories and accession number(s) can be found in the article/**Supplementary Material**.
