# Supplementary material for: Unraveling the role of GPCR signaling in metabolic reprogramming and immune microenvironment of lung adenocarcinoma: a multi-omics study with experimental validation
Source: Front Immunol. 2025 Jun 6;16:1606125. doi: 10.3389/fimmu.2025.1606125 (PMC12179119; doi:10.3389/fimmu.2025.1606125)
Supplement: Supplementary file 1 [file DataSheet1.zip › Supplementary Figures.DOCX]

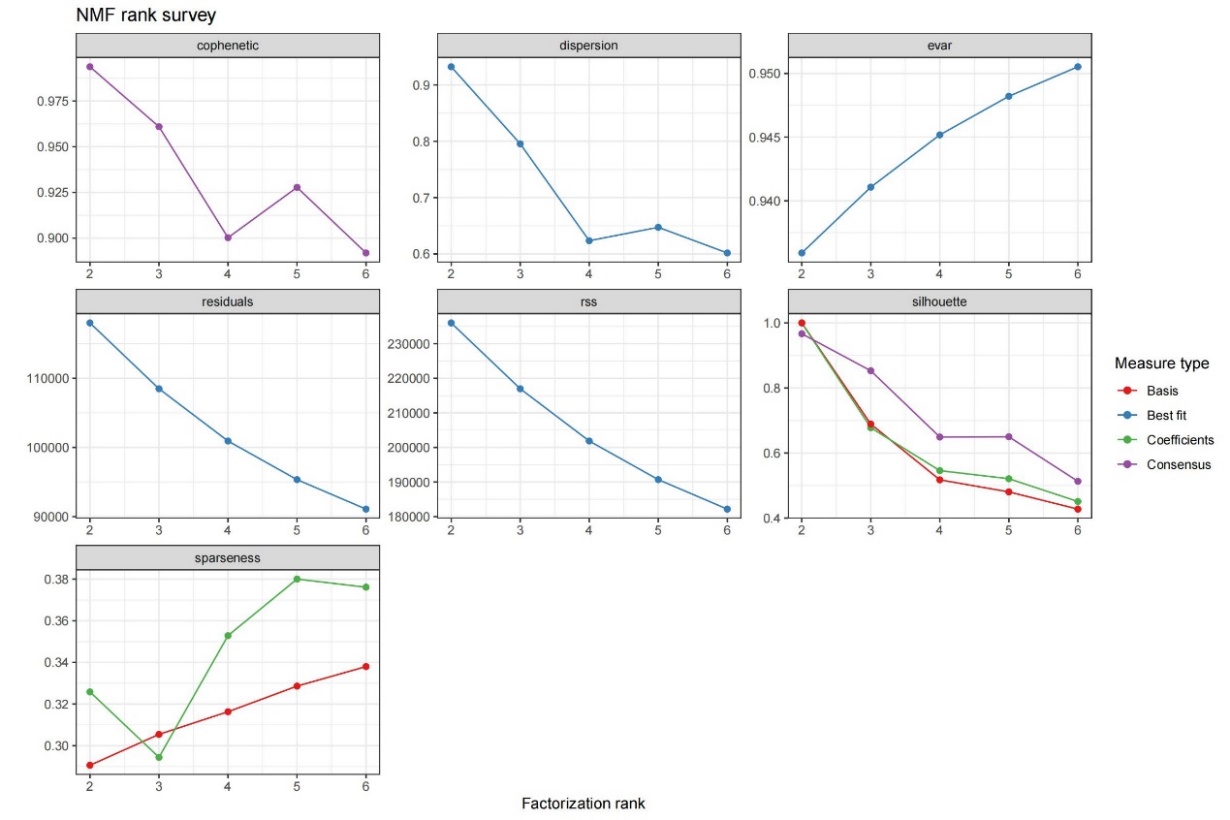


**Figure S1.** NMF rank survey.


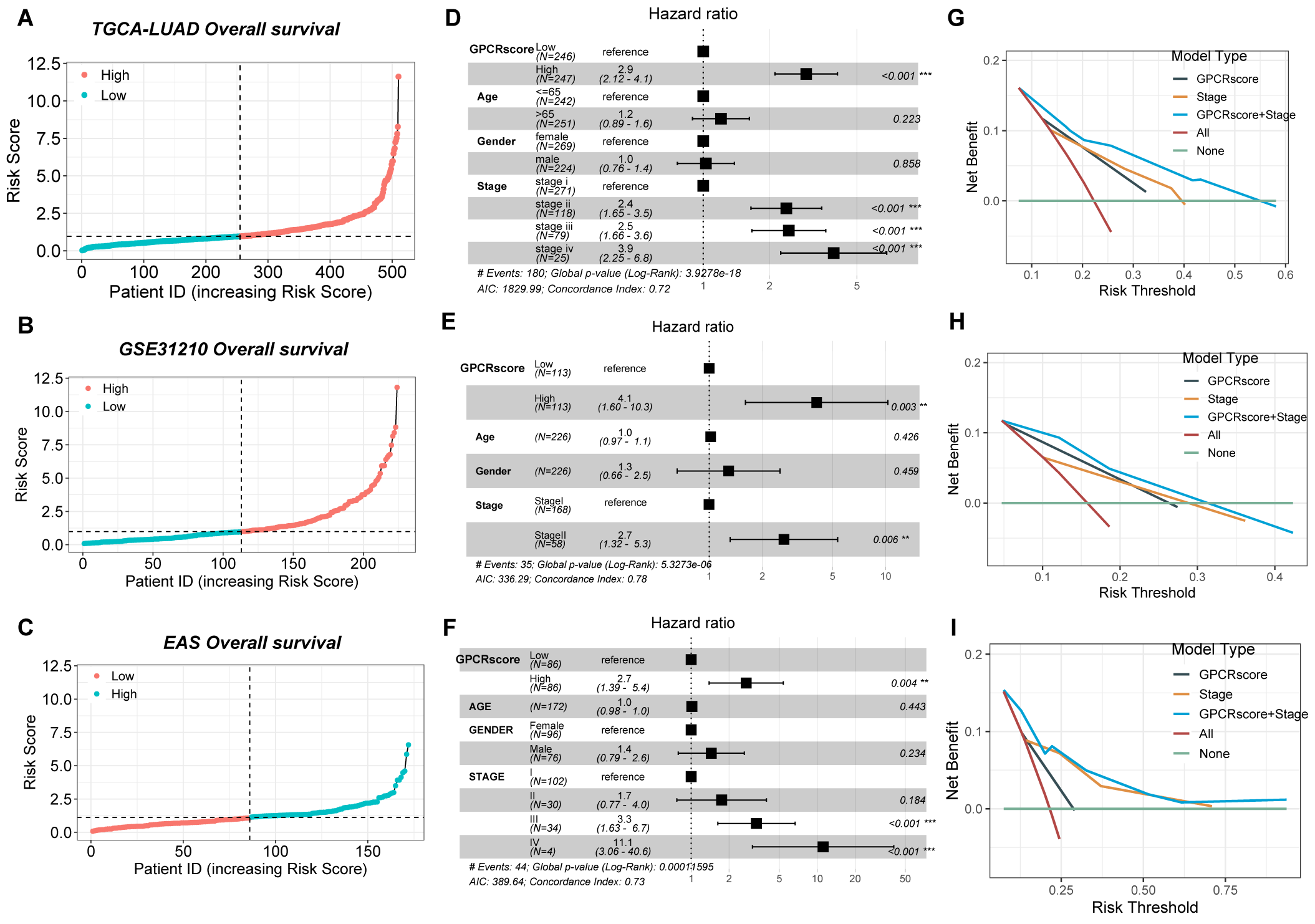


**Figure S2. Evaluation of the GPCR prognostic model across multiple datasets. (A-C)** Distribution of GPCRscore according to survival status and time in TCGA-LUAD, GSE31210, and EAS cohorts. **(D-F)** Multivariate Cox analysis of TCGA-LUAD, GSE31210, and EAS cohorts. **(G-I)** Decision curve analysis (DCA) showing the benefit by applying the GPCRscore and TNM stage.


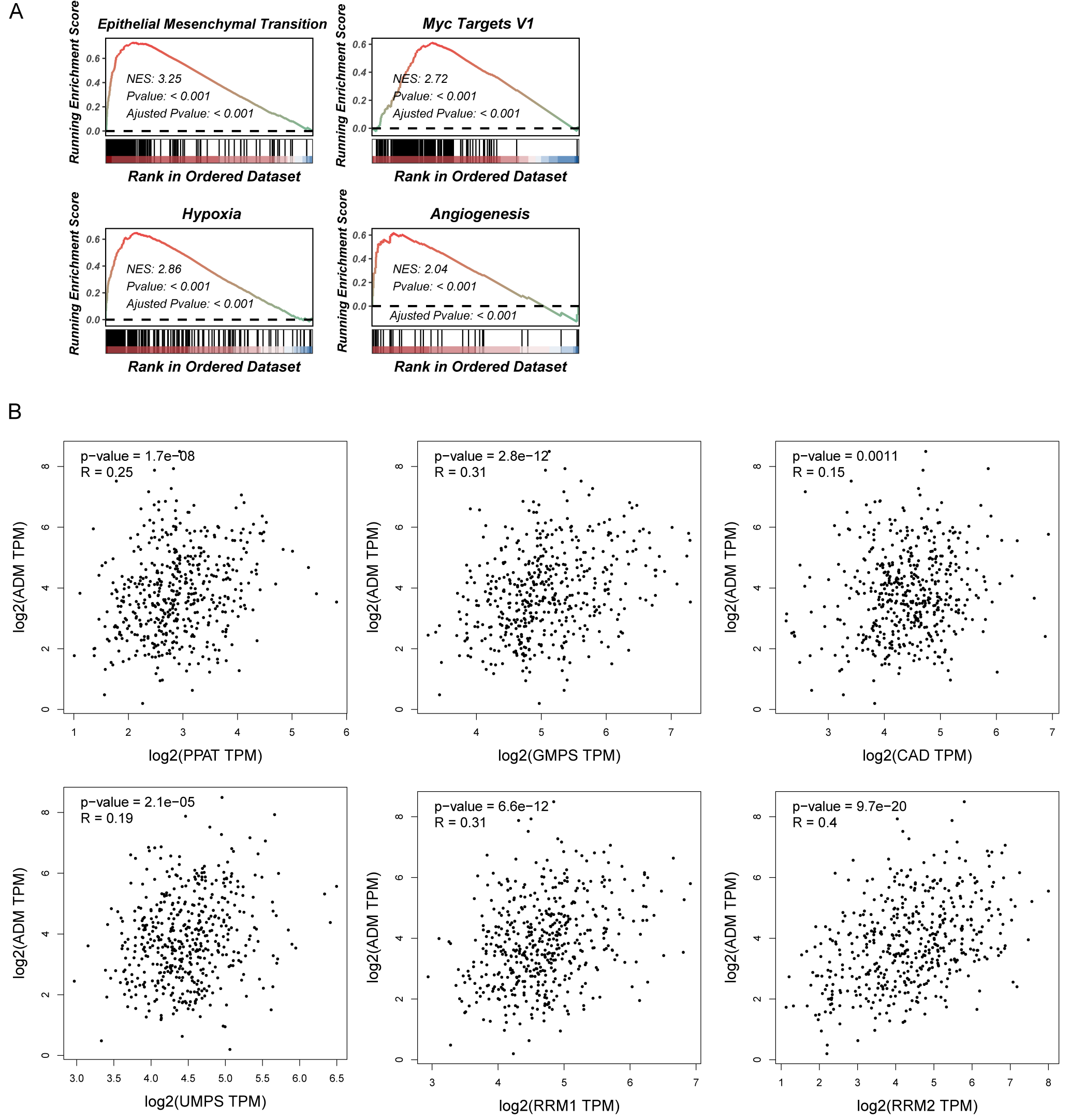


**Figure S3. The relationship between ADM and nucleotide metabolism. (A)** GSEA of DEGs between ADM-high and ADM-low showing the enrichment of Hallmark gene sets. **(B)** Scatter plot showing the correlation between ADM and purine (*PPAT*, *GMPS*, *RRM1*, and *RRM2*) and pyrimidine (***CAD*** and ***UMPS***) metabolic genes in TCGA-LUAD.


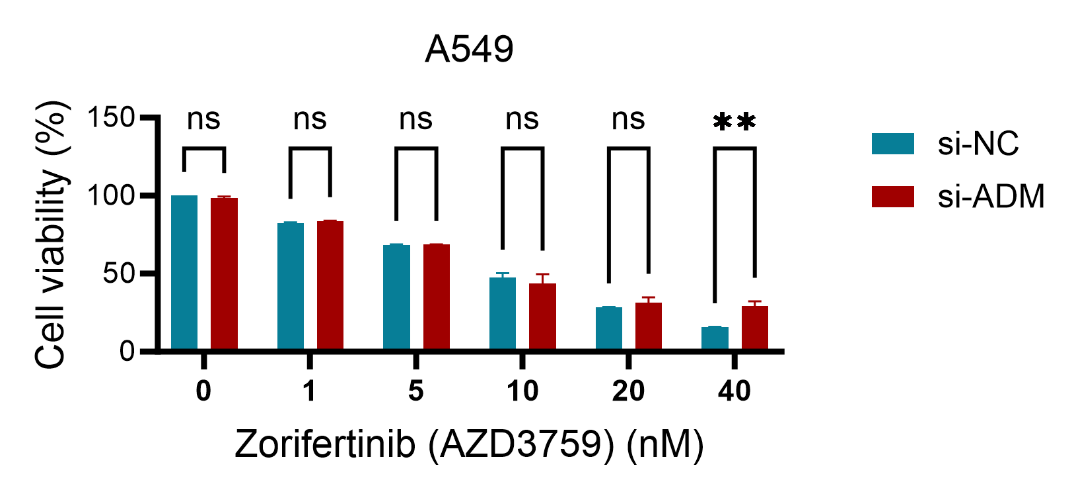


**Figure S4.** Barplot showing the relative viability of A549 cells treated with AZD3759 after si-ADM transfection, in comparison with the control group. Statistic tests: two-way ANOVA. Significance levels are denoted as *P<0.05, **P<0.01, ***P<0.001, ****P<0.0001.
